# Development of a Bayesian Network and Information Gain-Based Axis Dynamic Mechanism for Ankle Joint Rehabilitation

**DOI:** 10.3390/biomimetics10120823

**Published:** 2025-12-09

**Authors:** Huiguo Ma, Yuqi Bao, Jingfu Lan, Xuewen Zhu, Pinwei Wan, Raquel Cedazo León, Shuo Jiang, Fangfang Chen, Jun Kang, Qihan Guo, Peng Zhang, He Li

**Affiliations:** 1 The Academy of VR and Art, Jiangxi University of Software Professional Technology, Nanchang 330041, China; huiguo.ma@alumnos.upm.es (H.M.); baoyuqi@jxuspt.edu.cn (Y.B.); zhuxuewen@jxuspt.edu.cn (X.Z.); wanpinwei@jxuspt.edu.cn (P.W.); chenfangfang@student.usm.my (F.C.); siele51923@smc.edu.kg (J.K.); 2 Centro de Automática y Robótica UPM-CSIC, Escuela Técnica Superior de Ingeniería y Diseño Industrial, Universidad Politécnica de Madrid, Ronda de Valencia, 3, 28012 Madrid, Spain; raquel.cedazo@upm.es; 3 School of Information Engineering, Quanzhou Ocean Institute, Shishi City, Quanzhou 362700, China; 25024082004@stu.hqu.edu.cn; 4 Department of Design, Kyungpook National University, Daegu 41566, Republic of Korea; jshuo1024@knu.ac.kr; 5 School of Art and Design, Wuhan University of technology, Wuhan 430070, China; 349256@whut.edu.cn; 6 School of Arts and Design, Yanshan University, Haigang District, Qinhuangdao 066000, China; 2021327350@knu.ac.kr

**Keywords:** Bayesian information gain, dynamic axis matching, ankle rehabilitation, hybrid serial-parallel mechanism, kinematic analysis

## Abstract

In response to the personalized and precise rehabilitation needs for motor injuries and stroke associated with population aging, this study proposes a design method for an intelligent rehabilitation trainer that integrates Bayesian information gain (BIG) and axis matching techniques. Grounded in the biomechanical characteristics of the human ankle joint, the design fully draws upon biomimetic principles, constructing a 3-PUU-R hybrid serial–parallel bionic mechanism. By mimicking the dynamic variation of the ankle’s instantaneous motion axis and its balance between stiffness and compliance, a three-dimensional digital model was developed, and multi-posture human factor simulations were conducted, thereby achieving a rehabilitation process more consistent with natural human movement patterns. Natural randomized disability grade experimental data were collected for 100 people to verify the validity of the design results. On this basis, a Bayesian information gain framework was established by quantifying the reduction of uncertainty in rehabilitation outcomes through characteristic parameters, enabling the dynamic optimization of training strategies for personalized and precise ankle rehabilitation. The rehabilitation process was modeled as a problem of uncertainty quantification and information gain optimization. Prior distributions were constructed using surface EMG (electromyography) signals and motion trajectory errors, and mutual information was used to drive the dynamic adjustment of training strategies, ultimately forming a closed-loop control architecture of “demand perception–strategy optimization–execution adaptation.” This innovative integration of probabilistic modeling and cross-joint bionic design overcomes the limitations of single-joint rehabilitation and provides a new paradigm for the development of intelligent rehabilitation devices. The deep integration mechanism-based dynamic axis matching and Bayesian information gain holds significant theoretical value and engineering application prospects for enhancing the effectiveness of neural plasticity training.

## 1. Introduction

With global aging and rising rates of sports injuries and strokes, rehabilitation medicine faces increasing demand. The ankle joint, a critical load-bearing and mobility joint, directly affects gait and balance; its dysfunction can cause secondary musculoskeletal disorders [1]. However, most existing rehabilitation devices adopt fixed-axis, single-DOF designs, which cannot match the instantaneous motion axis of the ankle, leading to force-line deviation and limited adaptability across rehabilitation stages [2].

In terms of mechanical design, various parallel and hybrid mechanism solutions have been proposed by Wang, Y [3]. Parallel structures enable multi-DOF motion but have constrained workspaces [4]. Serial mechanisms provide large ranges but suffer from low rigidity and poor axis alignment [5]. Recently, Zhang, W investigated axis matching, yet existing methods remain limited to fixed-axis pre-alignment [6]. Recent research has emphasized mechanical design and motion-axis alignment in ankle rehabilitation trainers [7]. Dong, H. et al.’s 3-PRR design provides multi-DOF motion yet suffers from limited workspace [8]. The 3R serial platform provides wide motion yet low rigidity, leading to force deviation and secondary injuries [9]. Chen, X et al.’s PUU-R hybrid design improves compliance and safety [10]. Pyun, K et al.’s dual-axis hinge design lacks real-time adaptability [11]. Optical tracking systems for real-time joint center measurement and compensation are limited by their precision and latency in high-frequency training [12]. Tao, L et al. achieved sub-millimeter positioning accuracy in industrial robot joint calibration using the ICP algorithm, offering valuable insights for dynamic axis matching in rehabilitation devices [13]. Current ankle rehabilitation trainers, limited by workspace constraints, rigidity issues, and poor real-time adaptability, could benefit from high-accuracy algorithms like ICP—successful in industrial robot calibration—for dynamic axis matching.

In terms of control strategies, although surface EMG-based closed-loop control is increasingly used in rehabilitation devices for neuromuscular activation, its optimization objective remains largely limited to trajectory tracking. This narrow focus often neglects key requirements such as minimizing interaction forces and adapting to patient progress, ultimately restricting the effectiveness of individualized therapy [14]. The Bayesian approach, adept at handling uncertainty, shows strong potential in medical decision-making. It provides a systematic framework to quantify rehabilitation uncertainties, integrating prior knowledge with new data via probabilistic models to continuously update beliefs and optimize decisions—ideal for unpredictable patient responses in rehabilitation. The Bayesian approach excels in managing uncertainty, demonstrating strong potential for medical decision-making by systematically overcoming the limitations of EMG control [15]. To build priors for the smart ankle trainer, we gather EMG signals from patients with comparable ankle injuries during key ankle motions, statistically derive movement-specific neuromuscular priors, and apply mutual information to quantify how EMG features and trajectory errors jointly guide dynamic training optimization—enabling real-time, patient-tailored adjustments. However, in rehabilitation robotics, current research focuses predominantly on state estimation, rarely integrates with mechanism design, and lacks a closed-loop framework based on “uncertainty quantification–strategy optimization” [16].

To overcome these limitations, this study proposes an intelligent ankle rehabilitation trainer that integrates Bayesian Information Gain (BIG) with dynamic axis matching. The system combines a 3-PUU parallel mechanism with an R-type rotational module to achieve rigid–flexible synergy, mimicking natural joint biomechanics. Using EMG signals and trajectory errors to construct priors, it employs mutual information for dynamic strategy optimization, forming a closed-loop “demand-perception–strategy-optimization–adaptive-execution” framework. This BIG-driven approach enables personalized, adaptive rehabilitation aligned with neuroplasticity principles.

## 2. Literature Review

### 2.1. Applications of Bayesian Methods in the Field of Rehabilitation Robotics

Bayesian methods, known for their advantages in uncertainty handling, have been increasingly applied in medical decision-making and robotic control. In rehabilitation robotics, most studies have utilized Bayesian networks for patient state estimation and motion intent recognition [17]. For instance, Strelet, E et al. fused multimodal sensor data using a Bayesian network model to enable real-time classification of stroke patients’ movement intentions, significantly enhancing robotic assistance effectiveness [18]. Ma, H et al. developed a dynamic Bayesian network (DBN) to predict patients’ next movement trends and incorporated the prediction results into robotic control strategies, thereby achieving adaptive impedance modulation [19]. However, these studies primarily focus on state estimation and pattern recognition, lacking efforts to deeply integrate the Bayesian framework with mechanism design.

### 2.2. Applications of Information Gain in Intelligent Control

The information gain (IG), commonly used in feature selection and decision trees, has seen limited use in control strategy optimization. Recently, mutual information has been introduced to reinforcement learning and adaptive control, such as Li, S et al.’s algorithm for stable trajectory tracking in uncertain environments [20]. In rehabilitation robotics, few studies apply information gain to motion strategy optimization, mostly using it offline to evaluate EMG features for motion recognition [21]. There remains a lack of system-level research that employs information gain as a real-time, closed-loop decision optimization criterion integrated with mechanism design and dynamic matching. In this study’s intelligent ankle rehabilitation control framework, information gain’s core role is “feature priority screening”: by calculating information gain for each candidate feature, it identifies core features most useful for distinguishing ankle injury grades. This provides key inputs for building the subsequent Bayesian network model, reduces redundant feature interference in decision-making, ensures rehabilitation strategies focus on the most critical indicators for injury assessment and functional recovery, and improves decision efficiency and accuracy.

### 2.3. Exploratory Integration of Bayesian Information Gain and Axis-Matching

The integration of Bayesian information gain with dynamic axis matching is a growing frontier in rehabilitation robotics. Recent work incorporates mutual information into real-time control to quantify how motion strategies reduce system uncertainty. For instance, Asghar et al. developed a multi-DOF robot calibration method using dynamic Bayesian inference and mutual information. By online acquiring joint errors and EMG signals, they built a conditional entropy minimization objective. This approach addresses the ankle joint’s dynamic rotation center variation, overcoming fixed-axis design limits like force-line deviation. Combined with the ICP algorithm, it enables real-time, sub-millimeter accurate estimation of the ankle’s instantaneous axis and trajectory [22]. Additionally, Saeedi, B et al. applied the BIG metric to strategy optimization in a wrist rehabilitation robot, dynamically selecting assistive torques by maximizing information gain to match rapid convergence training modes with individualized rehabilitation rhythms [23]. These studies suggest that deeply integrating information-theoretic metrics from probabilistic models with geometric registration algorithms can simultaneously improve the accuracy of control decisions and the biomechanical compatibility of mechanism motion. However, systematic investigations in the context of complex ankle joint rehabilitation remain scarce.

**Figure 1:**
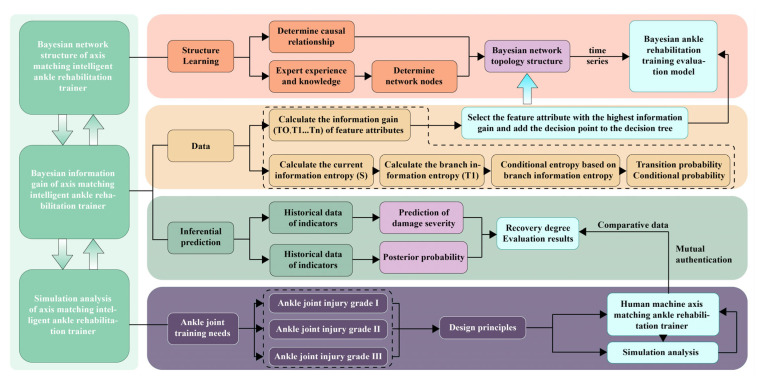
Bayesian Information Gain Research Framework Diagram of an Axis-Matching Intelligent Ankle Rehabilitation Trainer.

Although Bayesian methods have been applied in medical decision-making, most focus on state estimation and rarely integrate deeply with mechanical design. This study first combines Bayesian Information Gain (BIG) with dynamic axis matching, quantifying rehabilitation parameters’ uncertainty-reduction contribution to dynamically optimize training strategies. Our intelligent ankle trainer’s methodology (Figure 1) includes four interconnected modules: EMG acquisition, data processing, strategy generation, and feedback adjustment. This shifts ankle rehab from “mechanical adaptation” to “biomechanically intelligent adaptation,” aiming to enhance efficiency and compliance. Challenges remain in full-scene coverage and dynamic adaptability; future work must parallel algorithm refinement, hardware optimization, and clinical validation to translate lab tech to real-world “precision rehab for all.”

## 3. Bayesian Information Gain-Based Decision Optimization Model

### 3.1. Injury Level and Recovery Plan for Ankle Joint Patients

Ankle injuries manifest in various forms and can be classified into three levels based on severity [24]. Level I is characterized by mild pain, typically involving peripheral ligament damage without swelling. Level II involves moderate pain, with injuries including ligament and joint capsule tears accompanied by localized swelling [25]. Level III represents severe pain and the most serious damage, often involving ligament injuries combined with fractures, significant swelling, and potentially disuse muscle atrophy; see Table 1.

Rehabilitation requirements vary depending on the injury level. Functional recovery includes a range of motion training, muscle strength training, balance training, and proprioceptive training. Training modes include passive single-movement rehabilitation, active resistance rehabilitation, active gait simulation, and active free training. These modes should be selected according to the rehabilitation stage and specific needs: passive training is recommended in the early phase to promote circulation, followed by progressive adoption of strength-enhancing, gait-simulating, and free movement training. Given the variability in injury conditions and recovery progress among patients, personalized training ensures targeted interventions. Periodic training supports systematic continuity, and integrating multiple training modes can leverage their respective advantages to comprehensively meet the requirements of different recovery stages, thereby improving rehabilitation effectiveness.

### 3.2. Feature Selection and Information Gain Analysis

#### Data Sources and Variable Definitions

The research data were obtained from real clinical assessments of 100 patients with ankle joint injuries who participated in a six-week rehabilitation program using the prototype axis-matching trainer. The dataset was designed following a randomized mixed-disability-level structure, in which patients with Level I–III ankle disabilities were interspersed and evenly distributed across the full sample to avoid any predefined grouping or clustering artifacts. This design ensured statistical diversity and reduced sampling bias among different disability levels. For each patient, standardized clinical evaluations—including pain intensity (VAS), swelling diameter, joint stability, functional limitation, ankle range of motion (ROM), and surface electromyography (sEMG) signals—were collected at each assessment session to comprehensively characterize rehabilitation progression and neuromuscular response. Each record consists of the following five fields:

- Target Variable D: Disability Level, categorized into Level I, II, and III;
- Feature Set = {A1, A2, A3, A4}:
  A1: Pain Level (Visual Analog Scale, VAS);
  A2: Swelling Diameter (in cm);
  A3: Joint Stability;
  A4: Degree of Functional Limitation.

To ensure good model adaptability, the dataset incorporates a specially designed “weak feature correlation” mechanism. This includes scenarios such as “extensive swelling but low disability Level” and “mild pain but high disability Level,” effectively simulating the complex, nonlinear relationships between physiological indicators and functional states observed in real clinical settings.

According to Shannon’s information theory, the empirical entropy H(D) of the dataset D means ankle joint injury grade (i.e., disability grade) is used to quantify the uncertainty of disability levels. It is defined consistently with the information entropy of a random variable [26] from Equation (1):

(1) $$H(D)=-\overset{3}{\underset{k=1}{\sum }}\frac{\left|C_{k}\right|}{\left|D\right|}\log_{2}\frac{\left|C_{k}\right|}{\left|D\right|},$$

where C1, C2, and C3 represent the sets of samples for Level I, II, and III, respectively, and |C_k_| denotes the sample count in each category. For this study, the 40 patients include 13 Level I, 12 Level II, and 15 Level III cases. Substituting these into Equation (1) yields H(D) ≈ 1.58 bits.

Given a feature Aj with possible values {aj1, aj2, …}, the conditional entropy H(D|Aj) represents the uncertainty in disability level when the value of Aj is known, and is computed as Equation (2):

(2) $$H\left(D|A_{j}\right)=\underset{i}{\sum }P\left(A_{j}=a_{ji}\right)H\left(D|A_{j}=a_{ji}\right)$$

where H(D|Aj = aji) denotes the entropy of disability levels given that Aj takes the value aji. Based on these definitions, the information gain IG(D, Aj) measures the reduction in uncertainty before and after partitioning the dataset by feature Aj, and is calculated using Equation (3):

(3) $$IG(D,A_{j})=H(D)-H(D|A_{j}).$$

Table 2 presents the entropy and conditional entropy corresponding to each feature and its values, as well as the resulting information gain. The table shows that the degree of functional limitation (A4) yields the highest information gain (0.591 bits), indicating that it contributes the most to reducing the uncertainty in disability classification compared to the other features [27].

The classification target variable D (disability level: Level I–III—four prior features A were selected: pain level, swelling diameter, joint stability, and degree of functional limitation. Among them, swelling diameter and joint stability are important objective indicators of structural injury, while functional limitation reflects the outcome in terms of movement capability. These clinical indicators serve as prior input features for the Bayesian model and exhibit weak correlations with disability level.

As shown in the table, the degree of functional limitation has the highest information gain (0.591 bits), indicating the greatest contribution to reducing uncertainty in disability level classification. Thus, it is the most discriminative prior feature for disability-level determination. This approach enables the efficient selection of variables that contribute most to uncertainty reduction, thereby optimizing the structure learning process of the Bayesian network.

### 3.3. Bayesian Network Operations and Analysis

The information gain analysis, based on quantitative computation of four feature attributes—pain level, swelling diameter, joint stability, and degree of functional limitation—not only clarifies the differential contributions of each feature to disability level discrimination, with functional limitation identified as the core feature, but also reveals the underlying correlation patterns between feature attributes and the target variable (disability level). These patterns serve as key premises for constructing the Bayesian network. By establishing feature priorities and relational structures through information gain, the Bayesian network transforms the deterministic associations between features and disability levels into quantifiable probabilistic relationships, thereby constructing a reasoning framework capable of simulating the dynamic evolution from “feature attributes” to “disability classification”.

#### 3.3.1. Bayesian Network Structure and Variable Quantification

Based on the results of the information gain analysis, the degree of functional limitation was decomposed into four sub-dimensional nodes for quantification. These sub-nodes were determined through an in-depth analysis of the factors influencing the design of the intelligent ankle rehabilitation trainer and aligned with the fundamental theoretical framework of Bayesian networks. The node structure of the dynamic Bayesian network (DBN) was established using expert knowledge. The specific DBN node variables are as follows:

- Target Node Set = {Pain Control (G1), Tissue Repair (G2), Stability Improvement (G3), Load-Bearing Function Recovery (G4) → Achieved/Not Achieved}. These target nodes represent the overall recovery status of rehabilitation training and serve as core indicators for evaluating rehabilitation effectiveness.
- Second bullet; Intermediate Node Set = {Talocrural Joint Recovery Status (R1), Subtalar Joint Recovery Status (R2) → Recovered/Impaired}. These intermediate nodes reflect the recovery or damage status of the talocrural and subtalar joints during rehabilitation and are key factors influencing training outcomes.
- Node Set = {Flexion–Extension Function (Dorsiflexion/Plantarflexion) (B1), Inversion–Eversion Function (B2), Rotational Function (Internal/External Rotation) (B3), Stretching Function (B4) → Normal/Mildly Restricted/Severely Restricted, and Ankle Injury Level (P1) = Level I, II, III}. These observed nodes represent specific joint movements and injury levels that can be directly measured or assessed and are used to infer the states of intermediate and target nodes.

The clinical rationale for dependencies of directed edges between nodes stems from clinical patterns where more severe injuries correlate with a higher probability of restricted joint mobility—for example, the dependency “ankle joint injury grade (P1) → flexion–extension function (B1)”—and the dependency “subtalar joint repair status (R2) → stability improvement (G3)” is based on the direct impact of the subtalar joint’s structural integrity on ankle joint stability. It explains how core features selected via information gain screening—such as the degree of functional limitation (with an information gain of 0.591 bits)—determine node association priority: nodes tied to high-information-gain features (e.g., B1, B2) have higher connection weights with target nodes (G1–G4), reducing interference from redundant associations and preserving inference accuracy.

Table 3 presents the “prior probabilities of each node”, all derived from the baseline assessment results of 100 enrolled patients. Specifically, 22 patients (22%) had Grade I injury, and 53 patients (53%) did not meet pain control criteria before intervention; the prior probabilities of other nodes follow the same logic, reflecting the actual proportions of corresponding states in the sample. Table 4 outlines the “conditional probabilities of nodes”, calculated via grouped statistics from the 100 samples. Among the 28 patients with both the tibiotalar and subtalar joints repaired, 24 achieved pain control—this proportion 24/28 ≈ 0.85 serves as the corresponding conditional probability. All other conditional probabilities are constructed using the same “sample grouping—proportion meeting criteria” logic. For readability, probabilities are rounded to two decimal places in the tables, while the exact numerators and denominators are provided in the Supplementary Material.

Following the determination of the DBN node variables, the time slice interval was set to a single training session (30 min). The construction of Conditional Probability Tables (CPT)—a core component of the Bayesian network—adopts a hybrid approach integrating empirical clinical data, expert consensus, and simulation validation. In a Bayesian network, the distribution of node states directly affects the posterior probability inference of disability levels. Features with higher information gain are assigned greater weight, thus becoming key paths linking feature nodes to target nodes. This allows the network to more accurately infer the probability of each disability level based on feature states.

**Figure 2:**
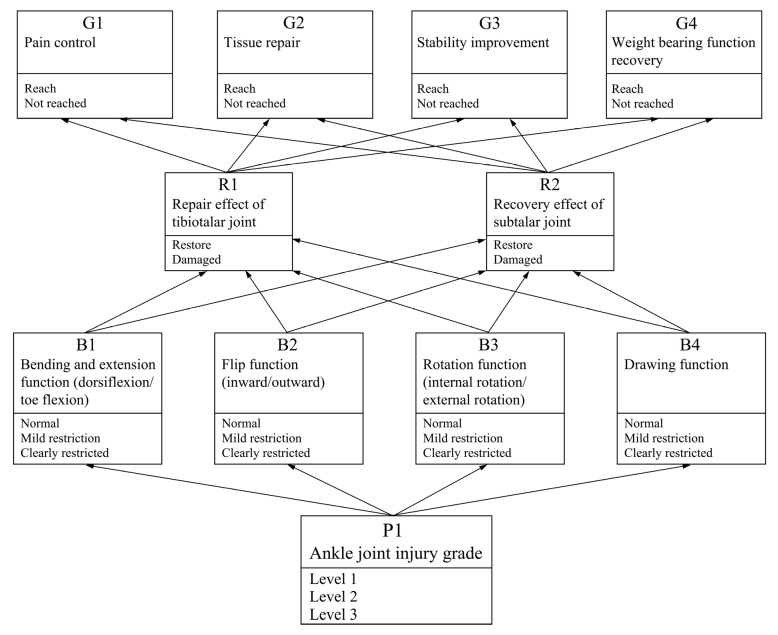
Bayesian Network Structure of the Axis-Matching Intelligent Ankle Rehabilitation Trainer.

Based on this structure, the study employed the professional Bayesian network construction tool GENIE, and, incorporating both the information gain analysis and variable quantification, developed the Bayesian network structure illustrated in Figure 2.

#### 3.3.2. Decision-Theoretic Optimization Objectives and Real-Time Posterior Probability Update Mechanism

- Decision-Theoretic Optimization Objectives

Two objectives are defined from a decision-theoretic perspective: first, to minimize classification uncertainty of ankle joint injury grades by filtering core features via BIG, thereby reducing mismatch loss between rehabilitation strategies and patient needs; second, to maximize the real-time probability of achieving core rehabilitation goals (e.g., pain control, weight-bearing function recovery) by leveraging highly relevant features retained by BIG, ensuring decisions focus on key priorities.

- Real-Time Posterior Probability Update Mechanism

Using a single training session (30 min) as a time unit, real-time feature and target node data are collected post-training to dynamically calibrate the Bayesian network’s Conditional Probability Table (CPT). Posterior probabilities are then iteratively updated in a hierarchical order: observation nodes → intermediate nodes → target nodes → injury grade nodes, enabling real-time optimization of decision-making.

#### 3.3.3. Methodological Details and Reproducibility of the Bayesian Model

To ensure model reproducibility, this section clarifies the model’s variable definitions, likelihood models, parameter learning methods, prior specifications, update equations, and core conditional probability tables—grounded in rehabilitation data from 100 ankle joint injury patients and the Bayesian network’s node structure.

- Likelihood model

All node states in the model follow a discrete multinomial distribution—i.e., for a given parent node state, the probabilities of all possible states of the child node sum to 1. The general form of the likelihood function is as follows:

Likelihood relationship between observation nodes (B1–B4) and intermediate nodes (R1–R2): P(Bj =b∣Rk =r)=θb∣r, where Bjdenotes an observation node, bits state value, Rkan intermediate node, rits state value, and θb∣rthe conditional probability parameter;

Likelihood relationship between intermediate nodes (R1–R2) and target nodes (G1–G4): P(Gm =g∣R1 =r1,R2 =r2 )=θg∣r1 ,r2, where Gmdenotes a target node, gits state value, and θg∣r1 ,r2 the conditional probability parameter.

- Uncertainty propagation

The proposed model propagates uncertainty analytically through the Bayesian network structure. All node states are modeled as multinomial variables, and their parameters are learned from data under conjugate Beta/Dirichlet priors. Given observed evidence, posterior distributions over intermediate and target nodes are obtained by applying standard Bayesian update rules and exact inference in the directed acyclic graph. In this study, closed-form updates are sufficient to capture uncertainty in the multinomial parameters and to compute posterior probabilities and credible intervals, without resorting to explicit Monte Carlo sampling. Sampling-based techniques such as Markov chain Monte Carlo or particle filters could be incorporated in future work for more complex network structures or continuous EMG features.

- Parameter Learning Methods and Overfitting Control

All model parameters (prior probabilities, conditional probabilities) are learned via maximum likelihood estimation (MLE), using baseline assessment data and post-training follow-up data from 100 patients:

- Prior probability learning (corresponding to Table 4): For root nodes without parent nodes (P1, B1–B4), their prior probability P(X = x) equals the frequency of node Xtaking state x in the sample, as shown in Equation (4):

(4) $$P(X=x)=\frac{Number\;of\;cases\;with\;X=x\;in\;the\;sample}{Total\;sample\;size\;\left(100\;cases\right)}$$

- Conditional probability learning (corresponding to Table 5): For child nodes with parent nodes (R1–R2, G1–G4), their conditional probability P(Y=y∣Parent(Y)=p) equals the frequency of child node Ytaking state y among samples where parent node(s) Ytake state p—i.e., P(Y = y \mid \text{Parent}(Y) = p) = \frac{\text{Number of cases with child node } Y = y \text{ among samples where parent node(s) } Y = p}{\text{Total number of cases where parent node(s) } Y = p}}.

The overall network structure is primarily determined by clinical expert knowledge about causal relations between injury level, functional limitation, joint repair and rehabilitation goals, and is further constrained by the feature ranking results of Bayesian information gain. This “expert-informed plus IG-filtered” design limits the search space and reduces the risk of overfitting that can occur in fully data-driven structure learning. To check generalization, the dataset is split into training and validation subsets; CPTs are learned on the training set and predictive performance for disability level and goal attainment is evaluated on the validation set, confirming that the learned probabilities are stable and not overly sensitive to small perturbations of the data.

- Posterior Probability Update Equations

Posterior probability updates follow a hierarchical logic of observation nodes → intermediate nodes → target nodes, with core equations presented below:

- Posterior Update for Observation Nodes

Observation nodes (B1–B4) have no parent nodes; their posterior probabilities are corrected based on the frequency of real-time training data, as shown in Equation (5):

(5) $$P(B_{j}=b\;|\;real-time\;data)=\frac{Number\;of\;cases\;with\;B_{j}=b\;in\;real-time\;training}{Total\;number\;of\;cases\;\;in\;\;real-time\;training}$$

- Posterior Update for Intermediate Nodes

Taking intermediate node R1 as an example (its parent nodes are B1–B4), its posterior probability is calculated using Bayes’ theorem and the law of total probability, as shown in Equation (6):

(6) $$\begin{array}{l} P(R1=r|B1=b_{1},B2=b_{2},B3=b_{3},B4=b_{4})\\ =\frac{P(B1=b_{1},B2=b_{2},B3=b_{3},B4=b_{4}|R1=r)\cdot P(R1=r)}{P(B1=b_{1},B2=b_{2},B3=b_{3},B4=b_{4})} \end{array}$$

where P(R1 = r) is the prior probability of R1 (Table 4); $P(B1=b_{1},\ldots ,B4=b_{4}|R1=r)$ is the joint likelihood of the observation nodes (B1–B4 are conditionally independent given R1, so it decomposes into $\prod_{j}^{4}=P(Bj=b_{j}\;|\;R1=r)$; the denominator is a normalization constant computed by $\sum_{r=0}^{1}=P(B1=b_{1},\ldots ,B4=b_{4}|R1=r)\cdot P(R1=r)$. The posterior update equation for R2 follows the same logic as R1.

- Posterior Update for Target Nodes

Taking target node G4 as an example (its parent nodes are R1 and R2), the posterior probability calculation Equation (7) is:

(7) $$P(G4=g\;|\;R1=r_{1},R2=r_{2})=\frac{P(R1=r_{1},R2=r_{2}|G4=g)\cdot P(G4=g)}{P(R1=r_{1},R2=r_{2})}$$

where P(G4 = g) is the prior probability of G4 (Table 4); P(R1=r1 ,R2=r2 ∣G4=g) is the joint likelihood of the intermediate nodes (parameters from Table 5); the denominator is a normalization constant computed by summing over g = 0.1: $\sum_{g=0}^{1}=P(R1=r_{1},R2=r_{2}\;|\;G4=g)\cdot P(G4=g)$. The posterior update equations for G1–G3 follow the same logic as G4.

#### 3.3.4. Node Status Scoring Criteria

To precisely evaluate the intervention effectiveness of the intelligent ankle rehabilitation trainer, this study conducted a six-week follow-up investigation involving 100 patients with ankle injuries. Multidimensional data collection was employed to ensure comprehensive and accurate information acquisition. During the data collection phase, observational methods were used to record the training conditions while patients used the intelligent ankle rehabilitation trainer. This included measurements of load intensity, activity frequency, real-time posture (e.g., flexion and inversion angles captured via motion capture systems), training duration, rest intervals, and immediate patient responses (such as pain feedback and degree of functional limitation). These observations were used to construct a behavioral database for ankle function training. In parallel, a questionnaire survey was designed based on the observed data, covering patient-reported experiences such as perceived force during training, self-assessed training difficulty, device-wearing comfort, and subjective evaluations of functional improvement. These supplemented the objective data by quantifying the user experience. Additionally, professional functional assessments were performed using joint range-of-motion measurement devices and EMG monitoring technologies. These tools collected precise data on ankle flexion–extension, inversion–eversion, rotation, and stretching functions, which were evaluated against clinical diagnostic criteria to assess joint recovery; see Supplementary Material.

For parameter optimization and outcome evaluation of the intelligent ankle rehabilitation trainer, a multidimensional quantification framework based on information gain analysis was developed using the GeNIe modeling environment (BayesFusion). The disability level (P1), identified as the core decision variable through information gain screening, was assigned the highest weight. Level III injuries (score: 5) directly determined the initial intervention plan and device calibration benchmark, triggering high-intensity intervention modes in the trainer. At the functional recovery node level, flexion (B1) and inversion (B2) were designated as core training targets (score: 5), addressed through dynamic angle correction modules for precision intervention. Rotation (B3) and stretching (B4) functions (score: 4) served as auxiliary recovery targets, to be gradually improved through progressive training, and were scored lower than the core functions. In terms of joint repair assessment, the talocrural joint (R1)—as the key load-bearing structure—was scored 5 when impaired and served as a critical index for adjusting load-bearing parameters of the trainer, directly reflecting its therapeutic effect. The subtalar joint (R2), responsible for supplementary stability, was assigned a score of 4 and adjusted via a lateral support module. In the rehabilitation goal system, load-bearing function recovery (G1, score: 5) was identified as the ultimate intervention target for the trainer, requiring full-cycle training from passive assistance to active load-bearing, directly reflecting the device’s value. Stability enhancement (G2, score: 4) was the necessary prerequisite, achieved through a balance control module. Pain control (G3, score: 3) and tissue repair (G4, score: 3) provided foundational support through biofeedback and analgesic modes. This scoring framework, with its hierarchical weighting and modular response mechanism, enables the intelligent ankle rehabilitation trainer to adaptively achieve foundational support via pain management and biofeedback, while prioritizing higher-order functional restoration goals; details are provided in Table 5.

In the quantitative evaluation system of the ankle rehabilitation trainer, unachieved states (e.g., G1 = 1, G2 = 1) are not assigned scores. This design stems from the framework’s focused orientation toward the positive intervention goals of the intelligent ankle rehabilitation trainer. The core logic behind this approach can be explained through the following three aspects:

- Goal Orientation:

The scoring system quantifies only the “achieved states,” assigning different weights (3–5 points) to each target node (e.g., G1–G4) to directly reflect their contribution to overall rehabilitation outcomes. For example, load-bearing function recovery is assigned the highest score, emphasizing its role as the ultimate goal. The unachieved state essentially represents a “deficiency requiring improvement.” Its function lies in offering a contrast for verifying the trainer’s intervention effect (e.g., a decrease in unachieved rate indicates effective device regulation), rather than conveying value through positive scoring.

- Bayesian Inference Requirements:

Within the Bayesian network framework, the probabilities of unachieved states (e.g., the posterior probability of G1 = 1) are dynamically computed based on prior data and conditional probability tables. Their analytical value lies in probability trends rather than absolute scores. For instance, if the probability of G1 = 1 drops from 52% to 37% after intervention, this change quantitatively reflects the improvement in pain control and sufficiently supports the evaluation of the device’s effectiveness without requiring additional scoring.

- Clinical Intervention Logic:

The core function of the intelligent ankle rehabilitation trainer is to facilitate the transition from “unachieved” to “achieved.” The scoring system, therefore, only needs to clarify which achieved states take priority (e.g., stability enhancement should be prioritized). The intervention priority of unachieved states is already implicitly represented by the corresponding weights of the achieved states (e.g., a score of 4 for G3 = 0 implies that intervention toward achieving G3 = 1 is a priority), thus avoiding redundant information that might interfere with decision-making in device regulation.

In summary, the exclusion of scores for unachieved states ensures that the quantitative framework remains focused on the positive intervention objectives of the intelligent ankle rehabilitation trainer. It also aligns with the Bayesian network’s core logic—favoring probabilistic inference over additive scoring—thereby enhancing the efficiency and precision of priority judgments in device modulation. The prior probability distributions of each node are shown in Table 3. The Posterior probability of each node are shown in Table 6. Compared with existing EMG signal-based methods, the BIG framework systematically optimizes the decision-making process and significantly enhances personalized rehabilitation outcomes.

**Figure 3:**
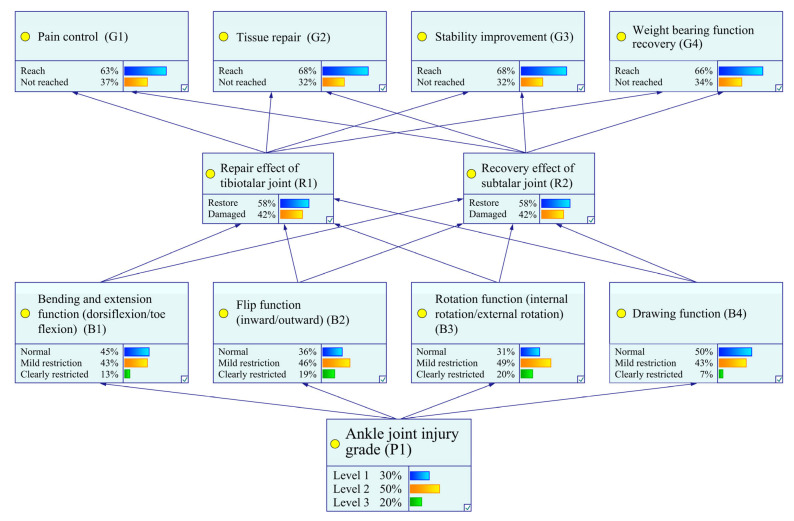
Nodestatus. Visualization of Node State Distributions in the Bayesian Network for Ankle Joint Rehabilitation.

Through multidimensional validation of prior, conditional, and posterior probabilities within a Bayesian framework, this study systematically demonstrates both the clinical value and theoretical soundness of the intelligent ankle rehabilitation trainer design, as shown in Figure 3. The main conclusions are as follows:

- Verification of Positive Improvement from Prior to Posterior Probabilities

The intervention of the intelligent ankle rehabilitation trainer significantly enhanced the achievement rates of core rehabilitation goals (G1–G4) and joint structural repair outcomes. For core objectives, pain control (G1) demonstrated a marked improvement from 38.0% to 62.9%, while tissue repair (G2) rose from 42.0% to 67.6%. Notably, load-bearing function recovery (G4) saw a dramatic surge from 20.0% to 65.8%. Concurrently, recovery rates for key joint structural nodes improved substantially: the talocrural joint (R1) progressed from 31.0% to 58.4%, and the subtalar joint (R2) increased from 31.0% to 57.7%. These findings highlight the trainer’s efficacy in driving measurable advancements across both functional and structural rehabilitation domains.

- Causal Validity Verification through Conditional Probability

Conditional probability analysis validated the clinical efficacy of the causal path-way “joint structural repair → functional recovery,” demonstrating that when both the talocrural joint (R1) and subtalar joint (R2) achieved recovered states (R1 = 0, R2 = 0), the achievement rates for all functional goals reached their peaks: 85.0% for pain control (G1), 92.0% for tissue repair (G2), 90.0% for gait stability (G3), and 95.0% for load-bearing function recovery (G4). These results were highly consistent with posterior intervention trends, where improvements in R1/R2 recovery rates correlated directly with synchronous enhancements in G1–G4 outcomes. This alignment not only confirms the design rationale of structural repair through mechanical correction modules but also underscores its profound compatibility with the pathophysiological mechanisms underlying ankle rehabilitation. The findings thereby establish a robust theoretical framework for the intervention mechanism, reinforcing the clinical relevance of targeted joint repair in driving functional recovery.

- Matching Validation between Injury Severity and Functional Recovery

For Grade III injuries (P1 = 2), the stepwise training protocol demonstrated clear efficacy. Posterior data show that the proportion of Grade III patients dropped to 20.0% from a high baseline, and their load-bearing function recovery (G4) achievement rate increased from 20.0% to 65.8%. These results confirm that the intelligent ankle rehabilitation trainer enables precise intervention for complex injuries through dynamic adjustment of training intensity and mechanical support parameters. This strongly supports the clinical applicability of the device’s “personalized intervention” design concept and confirms its capacity to accommodate the full spectrum of injury severity—from mild to severe.

In summary, this study, through a multidimensional probabilistic framework, systematically verifies the intelligent ankle rehabilitation trainer’s advantages in effectiveness, scientific rationality, and clinical adaptability. Using Bayesian information gain, we identified the core features that contribute most to discriminating ankle injury severity, which serve as key input variables for constructing the Bayesian network—thereby reducing interference from redundant features and improving the accuracy of injury-grade inference. At the same time, high-association features determined by information gain can directly inform the prioritization of functional capabilities in the mechanical design of the rehabilitation device, ensuring the design concentrates on ameliorating the principal injury-related functional impairments. Unlike standard Bayesian inference, which is limited to state estimation, the Bayesian Information Gain (BIG) framework introduces information gain as an optimization criterion. It reduces classification uncertainty via feature selection and drives dynamic adjustment of training strategies. This proactive optimization mechanism addresses the passivity of traditional probabilistic inference, significantly enhancing both the efficiency and accuracy of rehabilitation decision-making, and paves the way for advancing intelligent rehabilitation technologies toward precision-driven, full-cycle management.

## 4. Intelligent Ankle Rehabilitation Trainer Design and Evaluation

### 4.1. Motion Capture System Construction

#### 4.1.1. Structural Characteristics of the Ankle Joint

**Figure 4:**
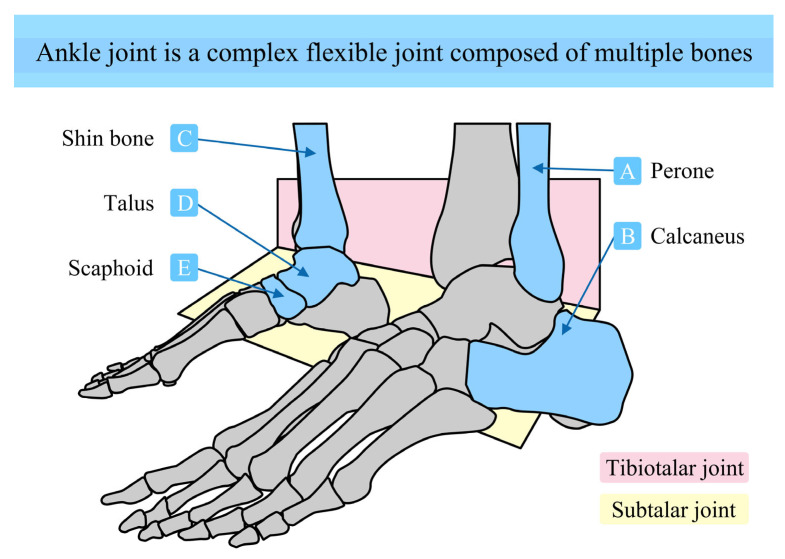
The ankle joint is a complex and flexible joint composed of multiple bones.

The ankle is a complex and flexible joint composed of several bones, including the tibia, fibula, talus, navicular, and calcaneus, and it contains key substructures such as the talocrural joint and subtalar joint, as shown in Figure 4. The tibia and fibula are the two long bones of the lower leg [28]. The tibia is located medially and is thicker, serving as the primary weight-bearing bone of the lower leg [29]. The fibula is situated laterally and is relatively slender. Arranged in parallel, they form the skeletal framework of the lower leg and provide superior bony support to the ankle joint [30]. The talus is located between the lower ends of the tibia and fibula and the upper parts of the calcaneus and navicular bones, serving as a central component of the ankle joint. It articulates superiorly with the tibia and fibula, and inferiorly with the calcaneus and navicular, functioning as a crucial bridge between the leg and the foot. The calcaneus is the largest tarsal bone in the foot, situated at the posterior and inferior part of the foot. The calcaneus forms the heel and supports body weight during stance. The navicular is a medially located tarsal bone positioned anterior to the talus. It forms a joint with the anterior aspect of the talus and contributes to both the mobility and stability of the foot, playing a key role in the medial longitudinal arch. The talocrural joint is formed by the inferior articular surface of the tibia and the superior articular surface of the talus. The distal end of the tibia flares out to form the medial malleolus, while the lateral malleolus is formed by the distal fibula. These structures conform to the trochlear surface of the talus. This anatomical configuration allows the talocrural joint to perform plantarflexion (downward movement of the foot) and dorsiflexion (upward movement of the foot). The subtalar joint consists of the inferior articular surface of the talus, the posterior articular surface of the calcaneus, and articular surfaces on either side. This joint enables inversion (turning the sole inward) and eversion (turning the sole outward), increasing the versatility and adaptability of foot movements, particularly during activities such as walking and running on uneven terrain. Additionally, the talonavicular joint, though not a primary motion joint, cooperates with the talocrural and subtalar joints to facilitate complex foot movements such as adduction and abduction.

During human activities such as walking, running, and jumping, the talocrural joint, subtalar joint, and talonavicular joint work in coordination to perform a wide range of complex ankle motions. For example, during walking, these joints coordinate sequentially to achieve smooth foot lifting, ground contact, and propulsion. This coordinated movement among the joints ensures smoothness and stability in foot motion. The tibia–fibula complex transfers body weight through the talus to the calcaneus and navicular while the navicular assists in load sharing and maintaining arch stability. These bones and joints of the ankle also provide shock-absorbing functionality. During movement, when the foot strikes the ground, the compressive interactions between joint surfaces—along with the elastic properties of surrounding soft tissues such as ligaments and tendons—help absorb and dissipate ground reaction forces. This reduces impact on the body and protects bones and joints from injury.

#### 4.1.2. 3-PUU-R Hybrid Serial–Parallel Rehabilitation Mechanism

- Mechanism Design Principles

The core function of the intelligent ankle rehabilitation trainer is to facilitate the transition from “unachieved” to “achieved.” Based on the anatomical axes of the talocrural and subtalar joints, the mechanism is designed to achieve three degrees of rotational freedom—namely plantarflexion/dorsiflexion, inversion/eversion, and internal/external rotation—in addition to one translational (stretching) degree of freedom, enabling composite ankle motion. The mechanism accommodates the full physiological range of ankle motion: Plantarflexion: 0–50°; Dorsiflexion: 0–30°; Inversion: 0–25°; Eversion: 0–15°; Internal rotation: 0–20°; External rotation: 0–50° [31], as shown in Table 7.

Through the coordinated control of parallel branches (PUU structure) and a rotational joint (R), the mechanism achieves compliant tracking along arbitrary spatial axes, allowing dynamic adaptation to the Ankle Joint Motion Axis (AJMA). The device features adjustable ankle height (accommodating foot lengths from 20 to 30 cm) and foot width (8 to 12 cm), with a simplified structure designed to reduce manufacturing costs. Based on an impedance control model, the stiffness and damping parameters of the mechanism are dynamically tuned to suit different rehabilitation phases—high-damping passive training in the early stage, and low-damping active training in the middle to later stages. The system uses real-time motion axis data from a motion capture system to adjust the mechanism’s posture, ensuring alignment of foot and shank motion axes, thereby reducing the risk of shear-related injuries.

- Structural Features of the 3-PUU-R Hybrid Serial–Parallel Mechanism for Human Motion Axis Matching

**Figure 5:**
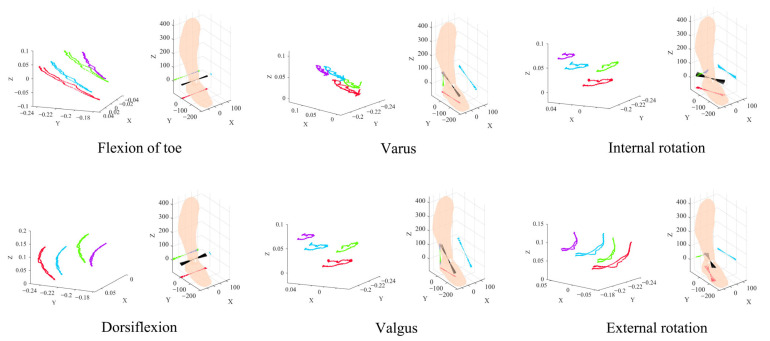
3D data trajectory and ankle joint motion axis diagram.

Based on six types of ankle rehabilitation motions (plantarflexion/dorsiflexion, inversion/eversion, internal/external rotation), an optical motion capture system was constructed using high-speed cameras, infrared reflective markers, calibration plates, tripods, and a central data processing unit. Markers were placed in a triangular region defined by the extensor digitorum brevis, anterior tibial tendon, and distal part of the extensor hallucis brevis. For leg positioning, the bony landmark located 20 cm vertically above the ankle center along the anterior tibial crest was used. Subjects performed six standard ankle rehabilitation exercises: plantarflexion/dorsiflexion, inversion/eversion, internal/external rotation, simulated writing, ball-stepping, and toe-standing. Each movement was repeated five times, lasting 10 s per repetition. Using Motive software, rigid body models of the foot and shank were constructed, and real-time pose data were streamed into MATLAB (R2024b, MathWorks). The final output included 3D trajectory data of the markers and the ankle joint motion axis diagram (see Figure 5), providing precise kinematic input for the axis-matching algorithm of the rehabilitation trainer.

**Figure 6:**
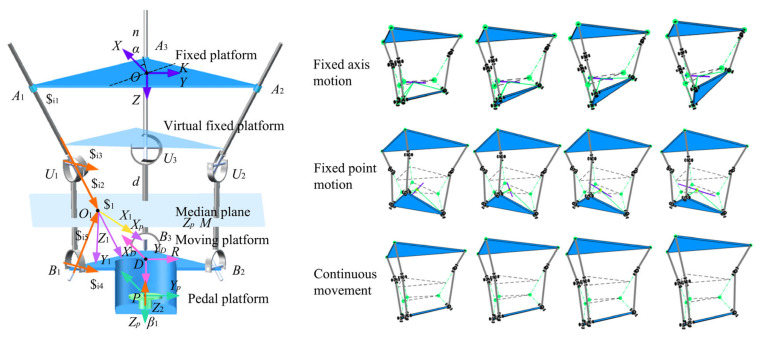
Kinematic Characteristics of the 3-PUU-R Hybrid Serial–Parallel Mechanism.

The schematic of the 3-PUU-R parallel ankle joint rehabilitation mechanism is shown in Figure 6. The mechanism consists of a fixed platform, a moving platform, three identical PUU branches, and a revolute pair on the moving platform. Each branch includes a prismatic joint (P) and two universal joints (U), with each U joint formed by two orthogonal revolute joints (R). This configuration provides the degrees of freedom required for ankle rehabilitation, covering the full range of motion, including plantarflexion/dorsiflexion, inversion/eversion, internal/external rotation, and traction. To capture the instantaneous variability of the ankle’s axis of rotation, the mechanism enables motion around arbitrary spatial axes. The arrangement of prismatic and universal joints ensures translational and rotational adaptability, while precision guides and crossed roller bearings maintain structural accuracy and compliance. Together, the joint combinations enable compound motion and provide a comprehensive basis for dynamic axis-matching rehabilitation.

The results of the Bayesian information gain analysis provide a direct guideline for prioritizing the mechanical and control design objectives of the 3-PUU-R mechanism. Among all clinical and functional features, the degree of functional limitation exhibits the highest information gain, followed by joint stability and swelling. These high-IG attributes are mapped onto functional target nodes (B1–B4) that the mechanism is designed to address: the four degrees of freedom and extended workspace enable comprehensive training in plantarflexion/dorsiflexion, inversion/eversion and axial rotation, with an additional translational degree of freedom for controlled stretching. In the impedance control scheme, stiffness and damping parameters are tuned with higher priority for motions that are most strongly associated with high-IG features (e.g., dorsiflexion and inversion in unstable ankles), so that the mechanical system and control strategy are explicitly aligned with the probabilistic feature priorities revealed by the BIG analysis.

### 4.2. Intelligent Ankle Rehabilitation Structural Design

**Figure 7:**
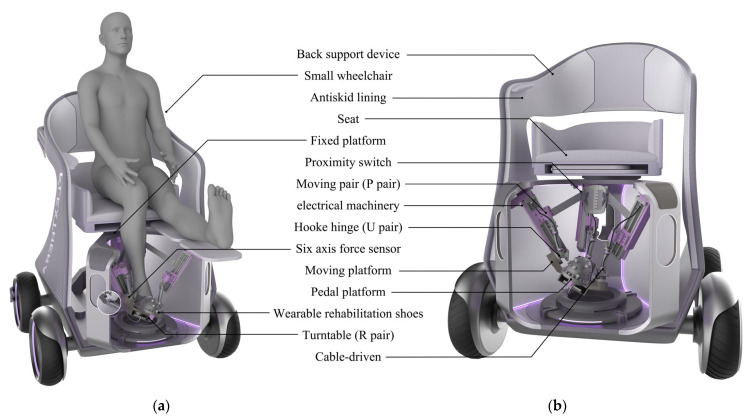
Mechanical structure model: (**a**) wearing effect; (**b**) structural composition.

The lower limb rehabilitation robot consists of fourteen components: a back support device, a compact wheelchair, a non-slip inner lining, restraining shell, fixed platform, proximity switch, prismatic joint (P joint), motor, Hooke joint (U joint), six-axis force sensor, moving platform, footplate platform, rotary joint (R joint), and wearable rehabilitation shoes. The mechanical structure model is shown in Figure 7.

**Figure 8:**
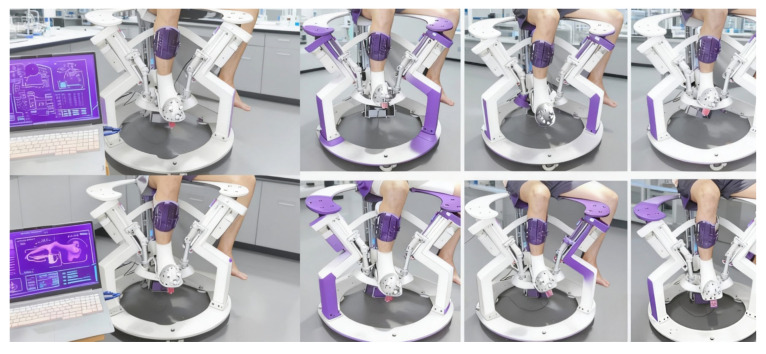
Functional Experimental Testing Figure of Prototype Machine.

Considering the patient’s ankle joint impairment and movement disorder, patients find it difficult to maintain balance during rehabilitation. Therefore, a small rehabilitation wheelchair with foot pads was designed to provide stable support during rehabilitation. The wheelchair is equipped with a back support device to assist the patient, and the seat support device has a lateral shifting function, allowing the patient to adjust more precisely to the ankle joint repair site from a sitting position. The wheelchair is equipped with lockable omnidirectional wheels, facilitating the movement of the entire device. Patients can undergo gait rehabilitation with the wheels unlocked or use other rehabilitation modes with the wheels locked, improving rehabilitation convenience without occupying additional space. The human ankle joint can be approximated as a ball-and-socket joint. Due to joint movement restrictions, when the ankle joint is constrained during rehabilitation, relying solely on the three translational degrees of freedom will inevitably cause discomfort. Therefore, two additional local degrees of freedom need to be incorporated into the ankle joint constraint to accommodate movement in the sagittal and coronal planes. To enhance overall rehabilitation comfort, two planes are used at the bottom of the foot to provide adequate support, matching the flexibility required for the arch of the foot during gait rehabilitation. A prototype has been developed for partial functions, conducting rehabilitation tests on patients with varying recovery needs across different phases based on their specific conditions. A digital twin system was established, integrating a virtual prototype with the physical rehabilitation robot to enable real-time monitoring of recovery speed and force magnitude during rehabilitation, as illustrated in the accompanying Figure 8.

## 5. Kinematic Analysis of the Rehabilitation Actuator

### 5.1. Degrees of Freedom of the 3-PUU-R Mechanism

**Figure 9:**
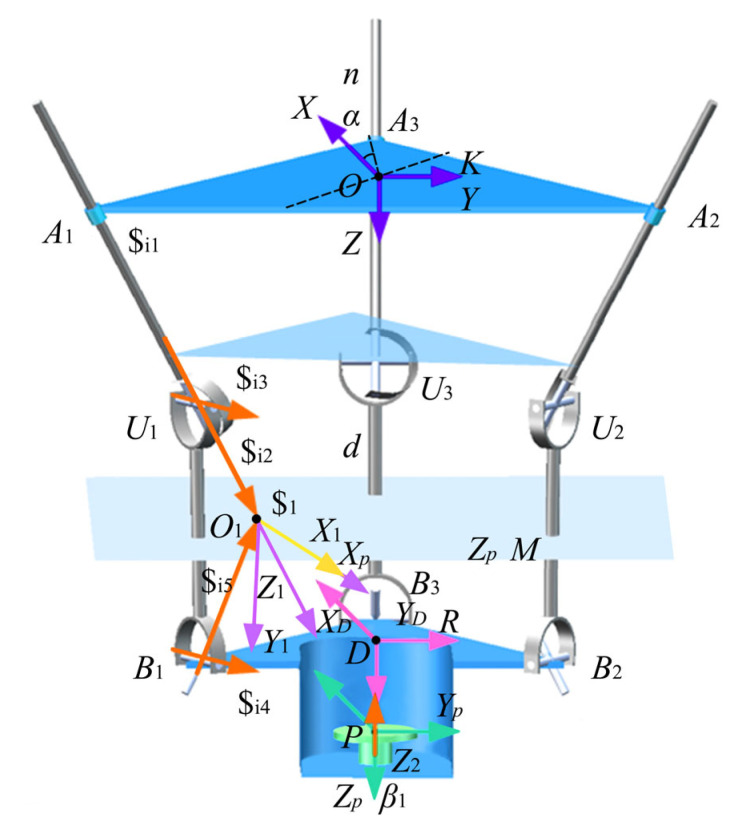
Schematic diagram of branch coordinate system.

The degrees of freedom (DOF) of the 3-PUU/R parallel ankle joint rehabilitation mechanism are analyzed below based on screw theory to ensure that the DOF meet the requirements of human ankle joint rehabilitation. Since the parallel section of the mechanism consists of three identical branches, one branch can be selected for analysis. A branch coordinate system is established as shown in the figure: the origin of the coordinate system for the *i*-th branch coincides with point $O_{i}$, the $X_{i}$-axis is parallel to the axis $r_{i3}$, the $Z_{i}$-axis is collinear with axis $r_{i2}$, and the $Y_{i}$-axis is determined according to the right-hand rule in Figure 9.

In the branch coordinate system $O_{1}-X_{1}Y_{1}Z_{1}$, the motion screw of the PUU branch can be expressed as Equation (8):

(8) $$\left\{\begin{array}{l} {\$}_{11}={\left(0\;\;0\;\;0;0\;\;0\;\;1\right)}^{T}\\ {\$}_{12}={\left(0\;\;0\;\;1;0\;\;0\;\;0\right)}^{T}\\ {\$}_{13}={\left(1\;\;0\;\;0;0\;\;e\;\;0\right)}^{T}\\ {\$}_{14}={\left(1\;\;0\;\;0;0\;\;0\;\;f\right)}^{T}\\ {\$}_{15}={\left(0\;\;m\;\;n;0\;\;0\;\;0\right)}^{T} \end{array}\right.$$

where the parameters e, f, m, n depend solely on the position of the revolute joints in the mechanism. According to relevant screw theory, the branch constraint screw system of the mechanism can be derived as Equation (9):

(9) $${\$}_{1}^{r}={\left(1\;\;0\;\;0;0\;\;0\;\;0\right)}^{T}$$

From this, it can be concluded that each branch of the 3-PUU parallel mechanism possesses a set of constraint screws. The constraint moments on the Hooke joints connected to the moving platform are perpendicular to the plane of the Hooke joints and act on the moving platform. Since these constraint moments are not coplanar, they are linearly independent. Therefore, analysis shows that the 3-PUU parallel mechanism has three degrees of freedom—two rotational and one translational. Because an additional revolute joint is mounted on the moving platform, the 3-PUU-R mechanism as a whole has four degrees of freedom.

The degrees of freedom can also be obtained using the modified Kutzbach–Grübler formula as Equation (10):

(10) $$M=6(\mathrm{n-g}-1)+\sum_{i=1}^{g}f_{i}+v$$

In the formula:

d—order of the mechanism (3 for planar mechanisms, 6 for spatial mechanisms);
n—total number of links, including the frame;
g—number of kinematic pairs;
$f_{i}$—degrees of freedom of the i-th kinematic pair;
v—overconstraints in the mechanism.

For the 3-PUU parallel mechanism: the total number of links is 8, the number of prismatic joints is 3, the number of Hooke joints is 6, the total number of kinematic pairs is 9, and there are no overconstraints (v = 0).

(11) $$\sum_{i=1}^{g}f_{i}=3\times 1+6\times 2\times 1=15$$

Substituting these values into Equation (6) yields:

(12) M=6(8−9−1)+15+0=3

Thus, the 3-PUU mechanism possesses 3 degrees of freedom.

### 5.2. Velocity Analysis of the 3-PUU-R Mechanism

**Figure 10:**
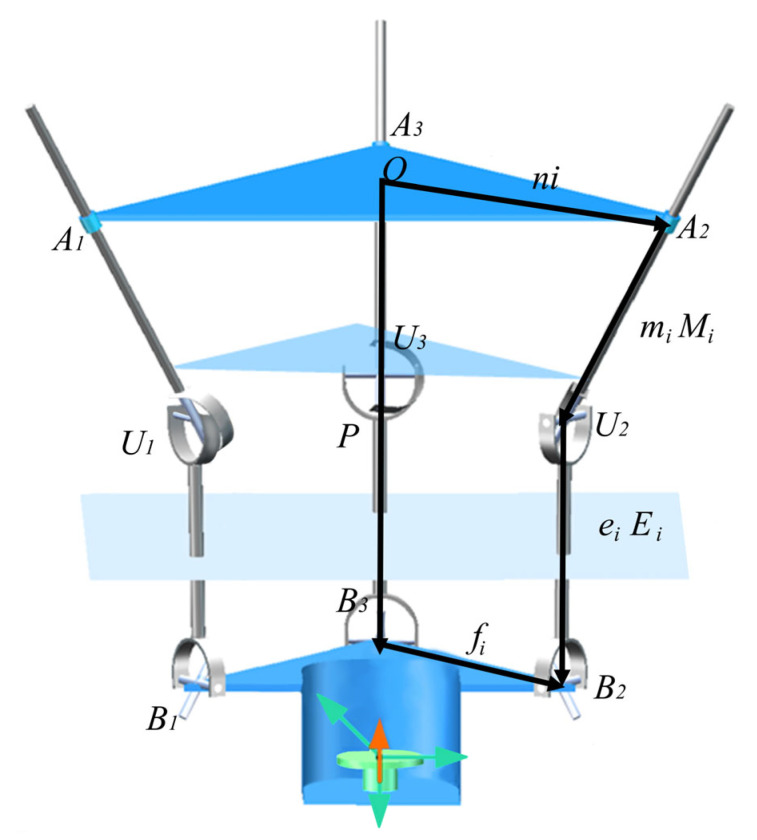
Vector closed-loop diagram.

According to Figure 10, the vector loop Equation (13) is written as:

(13) $$P+f_{i}=n_{i}+m_{i}M_{i}+e_{i}E_{i}$$

In the formula:

P—vector from the fixed platform to the moving platform;

$n_{i}$—vector from the center of the fixed platform to the center of point Ai;

$f_{i}$—vector from the center of the moving platform to the center of point bi;

$m_{i}$—stroke of the prismatic joint;

$M_{i}$—unit vector of the prismatic joint;

$e_{i}$—length of the ui bi rod;

$E_{i}$—direction vector of the uibi rod.

By differentiating both sides of the vector loop Equation (14) with respect to time, the expressions for the velocities can be obtained:

(14) $${\overset{\cdot }{V}}_{p}+w_{p}\times f_{i}={\overset{\cdot }{m}}_{i}M_{i}+e_{i}(w_{i}\times E_{i})$$

$\overset{\cdot }{V_{p}}={\left[\overset{\cdot }{P_{x}},\overset{\cdot }{P_{y}},\overset{\cdot }{P_{z}}\right]}^{T}$ represents the velocity of the moving platform, $w_{p}$ is the angular velocity of the moving platform, ${\overset{\cdot }{m}}_{i}$ represents the velocity of the prismatic joint, $w_{i}$ represents the angular velocity of the $U_{i}B_{i}$ rod.

Taking the dot product of both sides of Equation (14) with $E_{i}^{T}$, we obtain:

(15) $$(V_{p}+w_{p}\times f_{i})\cdot E_{i}^{T}={\overset{\cdot }{m}}_{i}M_{i}\cdot E_{i}^{T}$$

Expressed in matrix form, we have:

(16) $$J_{m}{\overset{\cdot }{m}}_{i}=J_{x}\left[\begin{array}{l} V_{p}\\ w_{p} \end{array}\right]$$

among which

(17) $$J_{x}=\left[\begin{array}{cc} {E_{1}}^{T} & -{E_{1}}^{T}{\overset{\wedge }{f}}_{1}\\ {E_{2}}^{T} & -{E_{2}}^{T}{\overset{\wedge }{f}}_{2}\\ {E_{3}}^{T} & -{E_{3}}^{T}{\overset{\wedge }{f}}_{3} \end{array}\right]$$

(18) $$J_{m}=\left[\begin{array}{ccc} M_{1}\cdot E_{1} & 0 & 0\\ 0 & M_{2}\cdot E_{2} & 0\\ 0 & 0 & M_{3}\cdot E_{3} \end{array}\right]\frac{1}{2}$$

where $\hat{f_{i}}$ denotes the skew-symmetric matrix of vector $f_{i}$, which can be expressed as:

(19) $${\overset{\wedge }{f}}_{i}=\left[\begin{array}{ccc} 0 & -f_{i}(3) & f_{i}(2)\\ f_{i}(3) & 0 & -f_{i}(1)\\ -f_{i}(2) & f_{i}(1) & 0 \end{array}\right]$$

Rearranging Equation (16) yields:

(20) $$m_{i}=J_{p}\left[\begin{array}{l} V_{P}\\ w_{p} \end{array}\right]$$

among which $J_{p}={\left[\begin{array}{cc} \frac{{E_{i}}^{T}}{M_{i}\cdot E_{i}} & \frac{-{E_{i}}^{T}{\overset{\cdot }{f}}_{i}}{M_{i}\cdot E_{i}} \end{array}\right]}_{\left(3\times 6\right)}$, and Equation (20) describes the mapping relationship between the driving velocities of the prismatic joints and the velocity of the moving platform in the 3-PUU parallel mechanism. Its Jacobian matrix depends only on the position of the moving platform.

The velocity *V_o_* at the origin O of the platform with series revolute (R) joints can be expressed as:

(21) $$V_{o}=V_{P}+w_{p}\times r$$

(22) $$w_{o}=w_{p}$$

where *r* denotes the revolute joint vector from the center of the moving platform to the center of the pedal platform, and *w_o_* denotes the rotational speed of the revolute joint reference point.

From Equations (21) and (22),

(23) $$\left[\begin{array}{c} V_{p}\\ w_{p} \end{array}\right]=\left[\begin{array}{cc} E_{3} & \overset{\wedge }{r}\\ 0 & E_{3} \end{array}\right]\left[\begin{array}{c} V_{o}\\ w_{o} \end{array}\right]$$

where $\hat{r}$ denotes the skew-symmetric matrix of vector *r*, and *E_3_* is the 3 × 3 identity matrix.

### 5.3. 3-PUU Acceleration Analysis

Taking the time derivative of both sides of Equation (20), we can obtain the relationship between the driving joint acceleration and the acceleration of the moving platform center:

(24) $$\begin{array}{l} a_{i}={\left(m_{i}\right)}^{’}=\frac{\left[\left(a_{p}+\varepsilon \times f_{i}+w_{p}\times (w_{p}\times f_{i}))\cdot E_{i}+(V_{p}+w_{p}\times f_{i})\cdot (w_{i}\times E_{i}\right)\right]\cdot (M_{i}\cdot E_{i})}{{\left(M_{i}\cdot E_{i}\right)}^{2}}\\ -\frac{\left[(V_{p}+w_{p}\times b_{i})\cdot m_{i2}\right]E_{i}\cdot \left[M_{i}\cdot (w_{i}\times E_{i})\right]}{\left(M_{i}\cdot E_{i}\right)} \end{array}$$

Differentiating Equation (22), we can obtain the angular acceleration $\varepsilon_{c}$ of the pedal platform as:

(25) $$\varepsilon_{p}=w_{p}’=w_{c}’=\varepsilon_{c}’$$

Differentiating Equation (21), we can obtain the linear acceleration $\xi_{c}$ of the pedal platform as:

(26) $$a_{p}=\xi_{c}-\varepsilon_{c}’\times r-w_{c}\times (w_{c}\times r)$$

### 5.4. 3-PUU RecurDyn Simulation Analysis

RecurDyn is a multi-body dynamics simulation software developed by Function Bay based on recursive dynamics theory. It is capable of simultaneously handling rigid and flexible body dynamics analyses and efficiently integrates the finite element method (FEM) to simulate complex rigid–flexible coupled systems. Its core advantages lie in using a relative coordinate system modeling and a recursive solution algorithm, which significantly enhances the speed and stability of solving dynamic equations. In this study, the 3-PUU mechanism was modeled dynamically using RecurDyn software. The dynamic model of the 3-PUU was established using RecurDyn 2025 software, with pre-configured constraint conditions including material properties of each component and friction contacts. By incorporating actual kinematic pairs, the rotational joint velocities were analyzed to verify the correctness and rationality of the structural design.

The simplified 3-PUU mechanism was imported into RecurDyn. In the assembled model, two mutually perpendicular rotational joints were set at each hinge to simulate realistic joint motion, along with prismatic joints. Displacement inputs were applied to the three prismatic joints, and the output angular velocity curves were used to assess the feasibility of the 3-PUU mechanism.

**Figure 11:**
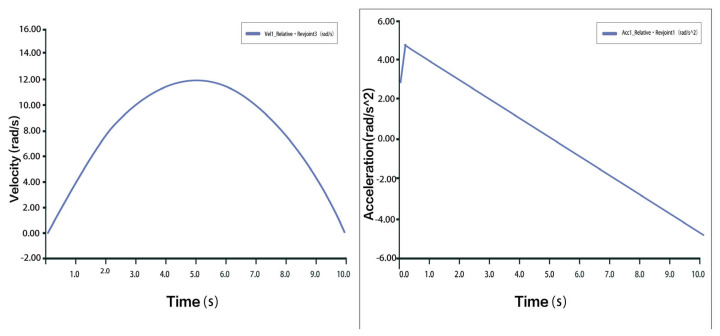
Velocity and Acceleration of Prismatic Joint A_3_ under Traction Movement.

**Figure 12:**
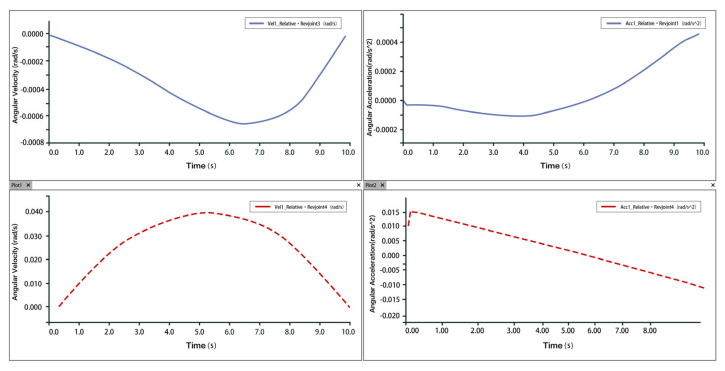
Velocity and Acceleration Curves of Hinge U_3_ under Traction Movement.

**Figure 13:**
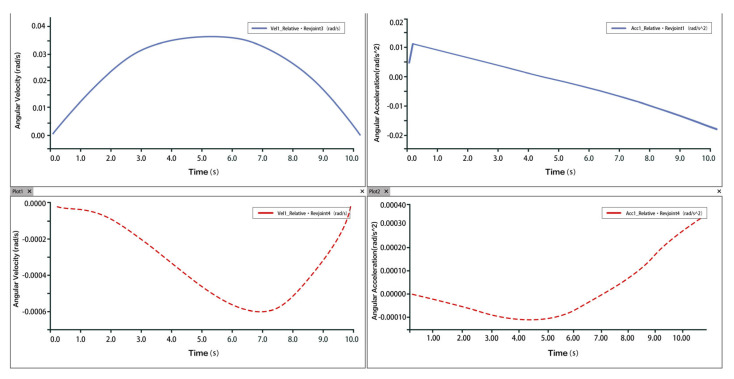
Velocity and Acceleration Curves of Hinge B_3_ under Traction Movement.

Taking one of the 3-PUU chains as an example, the moving platform is translated upward (traction motion). The velocity and acceleration of the prismatic joint (P joint) are shown in Figure 11. The velocity and acceleration curves of hinge U3 and hinge B3 are shown in Figure 12 and Figure 13, respectively. The results indicate that during the traction process, there is a sharp peak in the acceleration curve, likely caused by inevitable modeling or numerical errors within an acceptable range. Aside from this, the velocity and acceleration profiles are smooth and stable, consistent with mechanical motion laws, thus confirming the correctness and rationality of the 3-PUU mechanism during traction movement.

The ankle rehabilitation process involves inversion and eversion, internal and external rotation, stretching, and dorsiflexion and plantarflexion. Ankle dorsiflexion refers to the maximum flexion of the foot towards the shin, which is a fundamental function of the ankle joint. The normal range of motion for dorsiflexion is typically around 30°, and together with plantarflexion, constitutes the primary functional movement of the ankle. In this study, three displacement inputs related to the prismatic joints were provided, and the angular velocity and angular acceleration of the hinges were obtained through RecurDyn simulation to analyze the velocity–acceleration relationship of the 3-PUU mechanism.

**Figure 14:**
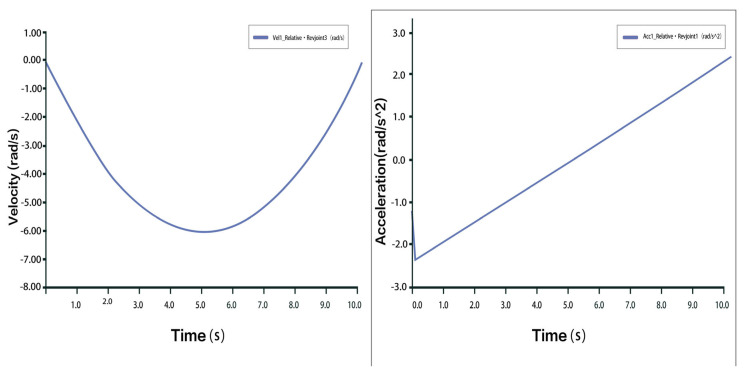
Velocity and Acceleration of Prismatic Joint A3 under Dorsiflexion Movement.

**Figure 15:**
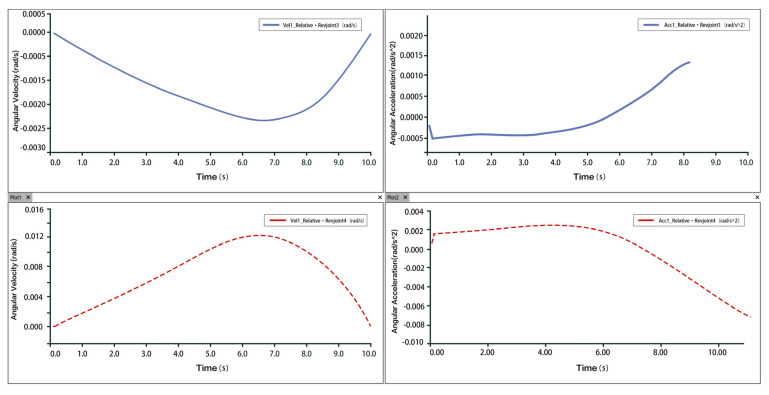
Velocity and Acceleration of Hinge U_3_ under Dorsiflexion Movement.

**Figure 16:**
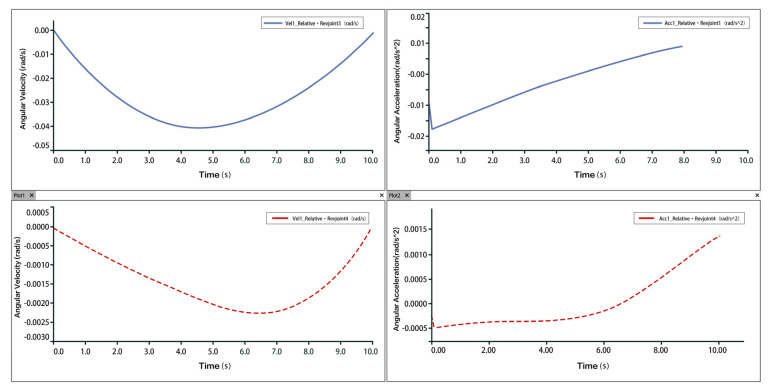
Velocity and Angular Velocity of Hinge B_3_ under Dorsiflexion Movement.

Taking dorsiflexion as an example, analysis of the velocity and acceleration plots shows that the peak velocity is approximately 6 mm/s, and the peak acceleration is about 2.3 mm/s^2^. The slope of the velocity curve represents the acceleration, and the correspondence between the velocity and acceleration plots indicates that the two rotational and one translational degrees of freedom of the 3-PUU mechanism meet the functional requirements for dorsiflexion movement. The velocity and acceleration of the prismatic joint (P-joint) are shown in Figure 14, those of Hinge U3 in Figure 15, and those of Hinge B3 in Figure 16.

**Figure 17:**
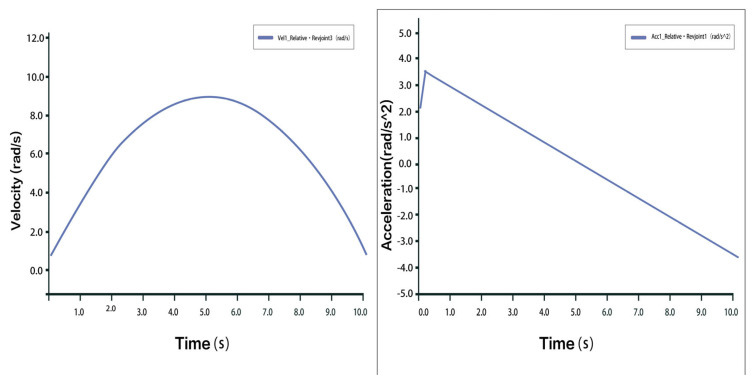
Velocity and Acceleration of Prismatic Joint A_3_ under Plantarflexion Movement.

**Figure 18:**
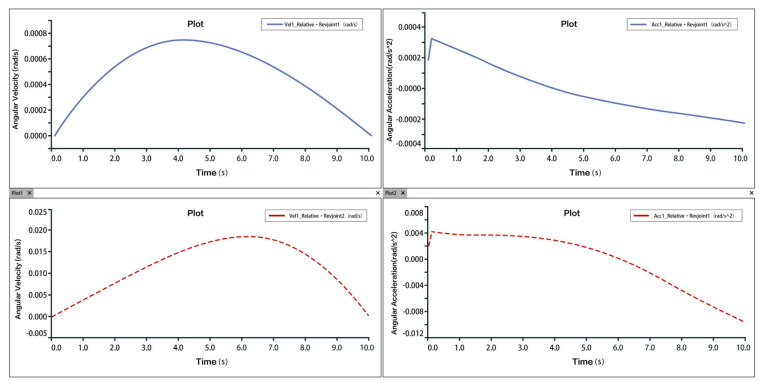
Velocity and Acceleration of Hinge U_3_ under Plantarflexion Movement.

**Figure 19:**
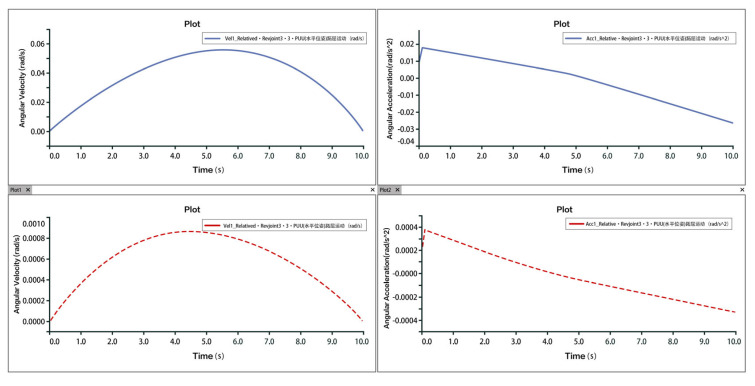
Velocity and Acceleration of Hinge B_3_ under Plantarflexion Movement.

Taking plantarflexion as an example, analysis of the velocity and acceleration curves indicates that the maximum velocity reaches approximately 9 mm/s, while the maximum acceleration is around 3.5 mm/s^2^. The slope of the velocity curve represents the acceleration, and the correspondence between the velocity and acceleration plots confirms that the two rotational and one translational degrees of freedom of the 3-PUU mechanism meet the functional requirements for plantarflexion movement. The velocity and acceleration of the prismatic joint (P-joint) are shown in Figure 17, those of Hinge U3 in Figure 18, and those of Hinge B3 in Figure 19.

Taking the analysis of velocities and accelerations during traction, dorsiflexion, and plantarflexion movements as an example: During the execution of the predetermined trajectory, the velocities and accelerations of each branch are uniform and stable, conforming to mechanical motion principles. Although minor errors exist, these primarily arise from unavoidable motion coupling during virtual prototyping simulation. These errors fall within a reasonable range, verifying the correctness of the dynamic model of the 3-PUU/R mechanism. The 3-PUU features two rotational and one translational degrees of freedom, which meet the requirements of ankle joint rehabilitation. Therefore, the rationality of the 3-PUU/R mechanism is confirmed.

## 6. Discussion

The design of the axis-matching-based intelligent ankle joint rehabilitation trainer proposed in this study demonstrates significant innovation and clinical application value compared to traditional devices. Unlike fixed-axis or single-DOF structures, which restrict movement to a single plane and cannot adapt to patient-specific conditions, the proposed 3-PUU-R mechanism provides four degrees of freedom and expands ROM coverage to over 95%. Simulation results indicate that real-time axis matching was associated with reduced localized stress concentrations and improved biomechanical compatibility; however, these findings are preliminary and require experimental validation. A benchmarking analysis against fixed-axis, single-DOF, and 3-PUU mechanisms further highlights these advantages. While conventional devices cover less than half of the ankle’s ROM and lack adaptive control, and 3-PUU designs extend ROM to 70–80% but remain limited by missing degrees of freedom and simple trajectory tracking, the proposed system achieves both structural completeness and dynamic adaptability. The integration of Bayesian Information Gain further strengthens this approach. By quantifying the contribution of rehabilitation parameters and dynamically optimizing training strategies, the system forms a closed-loop of “data–model–training” Compared with adaptive control based on fixed rules, the Bayesian Information Gain (BIG) framework achieves real-time optimization of strategies through mutual information maximization and prioritizes high-contribution features. This not only reduces overfitting but also enhances decision-making efficiency, supporting intelligent, personalized adaptation beyond the fixed-mode, passive-response strategies of conventional devices. Nevertheless, this study has limitations. Current validation is based primarily on kinematic simulations and data from healthy subjects or mildly impaired patients. Complex conditions such as severe stiffness, neuromuscular disorders, or early postoperative recovery remain untested. Future work should incorporate multi-center clinical datasets, refine the algorithm, and develop hardware prototypes to ensure robustness and clinical applicability. Ultimately, this paradigm of combining axis matching with Bayesian decision-making may enable a transition from mechanical adaptation to biomechanical intelligent adaptation, supporting full-cycle rehabilitation management.

## 7. Conclusions

Based on the biomechanical characteristics of the ankle joint, this study utilizes the Bayesian Information Gain framework by integrating the feature selection ability of information gain with the probability updating mechanism of Bayesian statistics, forming a systematic feature optimization and classification method. On this basis, a 3-PUU-R serial–parallel hybrid mechanism aligned with the ankle joint motion axis was constructed to achieve a balance between high stiffness and adequate compliance. The mechanism design ensures the consistency between the mechanical rotation center and the ankle joint’s rotation axis, further employing dynamic matching of the motion axis to improve comfort and safety issues caused by force line deviation during training. Additionally, prior distributions were constructed using surface electromyography and motion trajectory errors, establishing an uncertainty quantification decision model based on Bayesian Information Gain. The training parameters are dynamically adjusted, and the training objectives are optimized through a mutual information maximization strategy. Multi-posture simulations based on a three-dimensional digital human model verified the motion feasibility and human factor adaptability of the system. A closed-loop control architecture, “Demand Perception–Strategy Optimization–Execution Adaptation,” is proposed, where surface electromyography signals and trajectory errors are used to perceive the patient’s needs in real time, and Bayesian Information Gain drives the adaptive update of the training strategy. Compared to existing rehabilitation equipment, this design balances the compliance of the mechanism with its biomechanical adaptability. The cable-driven structure offers advantages such as light weight and adjustable working space, allowing it to accommodate the patient’s natural movement. Meanwhile, the 3-PUU-R serial–parallel hybrid mechanism provides comprehensive motion capabilities while maintaining the overall stiffness of the mechanism, refer to Appendix A for specific comparison. The Bayesian Information Gain method is introduced for the first time in optimizing rehabilitation training, integrating probability modeling with rehabilitation decision-making, and providing a new theoretical basis for the development of personalized rehabilitation strategies. A natural randomization experiment on disability grades was conducted with 100 participants to verify the validity of the design results. Comprehensive kinematic analysis and simulation evaluations indicate that the system meets the design requirements in terms of range of motion, force transmission, and safety, significantly improving patient engagement and training outcomes. Experimental results are consistent with the literature; the adaptive Bayesian-optimized training scheme shows a clear advantage over fixed schemes. In summary, this exploratory study integrates dynamic axis matching with a Bayesian information-gain-based optimization framework. Proof-of-concept simulations suggest improved alignment between training strategies and patient-specific biomechanics; definitive clinical benefits remain to be established in bench and multi-center studies.

## Figures and Tables

**Table 1:** Classification of ankle joint injuries.

Damage Level	Pain Level	Damage Situation	Swelling Condition
Level I	Mild pain	Peripheral ligament injury	No swelling
Level II	Moderate pain	Laceration of ligaments, joint capsules	Local swelling
Level III	Severe pain	Ligament injury combined with fracture	Significant swelling

**Table 2:** Statistical results of information entropy, conditional entropy, and information gain for feature values.

Characteristic Attribute (A_j_)	Attribute Value (A_ji_)	Value Information Entropy (Bit)	Attribute Conditional Entropy (Bit)	Information Gain IG (D, A_j_) (Bit)
Pain level (A_1_)	Mild (A_11_)	1.385	1.428	0.064
	Moderate (A_12_)	1.512		
	Severe (A_13_)	1.436		
Pain level (A_2_)	Limitations (A_21_)	1.402	1.395	0.097
	Medium (A_22_)	1.425		
	Widely (A_23_)	1.328		
Joint stability (A_3_)	Stable (A_31_)	1.086	1.152	0.340
	Basic stability (A_32_)	1.418		
	Unstable (A_33_)	0.935		
Joint stability (A_4_)	Mild (A_41_)	0.768	0.901	0.591
	Moderate (A_42_)	1.285		
	Severe (A_43_)	0.683		

**Table 3:** Prior Probabilities of Bayesian Network Nodes. (Calculated via frequency statistics of 100 patient clinical data and Beta distribution fitting.)

Node Name	State	Probability
Ankle joint injury grade P1	0	0.22
Bending and extension function B1	1	0.38
Flip function B2	2	0.4
Rotation function B3	0	0.28
Drawing function B4	1	0.52
Repair condition of tibiotalar joint R1	2	0.2
Repair condition of the subtalar joint R2	0	0.18
Pain Control G1	1	0.53
Organizational repair G2	2	0.29
Stability improvement G3	0	0.19
Weight bearing function recovery G4	1	0.56

**Table 4:** Conditional probability distribution of nodes.

Repair Condition of Tibiotalar Joint R1	Repair Condition of The Subtalar Joint R2	Pain Control G1		Organizational Repair G2		Stability Improvement G3		Weight Bearing Function Recovery G4	
		Achieve	Not Achieved	Achieve	Not Achieved	Achieve	Not Achieved	Achieve	Not Achieved
0	0	0.85	0.15	0.92	0.08	0.9	0.1	0.95	0.95
	1	0.6	0.4	0.7	0.3	0.65	0.35	0.85	0.85
1	0	0.6	0.4	0.6	0.4	0.75	0.25	0.5	0.5
	1	0.3	0.7	0.3	0.7	0.25	0.75	0.12	0.12

**Table 5:** Node status. Definition of Node States, Hierarchical Classification, and Factor Scoring Criteria in the Bayesian Network for Ankle Rehabilitation.

Node Name	Status Value	Status Level	Factor Rating
Ankle joint injury Level P1	0	Level Ⅰ	2
	1	Level Ⅱ	3
	2	Level Ⅲ	5
Bending and extension function B1	0	Normal	1
	1	Mild restriction	3
	2	Clearly restricted	5
Flip function B2	0	Normal	1
	1	Mild restriction	3
	2	Clearly restricted	5
Rotation function B3	0	Normal	1
	1	Mild restriction	2
	2	Clearly restricted	4
Drawing function B4	0	Normal	1
	1	Mild restriction	2
	2	Clearly restricted	3
Repair condition of tibiotalar joint R1	0	Restore	2
	1	Damaged	5
Repair condition of the subtalar joint R2	0	Restore	2
	1	Damaged	4
Pain Control G1	0	Restore	3
	1	Damaged	—
Organizational repair G2	0	Achieve	3
	1	Not achieved	—
Stability improvement G3	0	Achieve	4
	1	Not achieved	—
Weight bearing function recovery G4	0	Achieve	5
	1	Not achieved	—

**Table 6:** Posterior probability of each node.

Node Name	State	Probability
Ankle joint injury grade P1	0	0.3
	1	0.5
	2	0.2
Bending and extension function B1	0	0.445
	1	0.43
	2	0.125
Flip function B2	0	0.355
	1	0.455
	2	0.19
Rotation function B3	0	0.315
	1	0.485
	2	0.2
Drawing function B4	0	0.495
	1	0.434
	2	0.071
Repair condition of tibiotalar joint R1	0	0.584
	1	0.412
Repair condition of tibiotalar joint R1	0	0.577
	1	0.423
Pain Control G1	0	0.629
	1	0.371
Organizational repair G2	0	0.676
	1	0.324
Stability improvement G3	0	0.676
	1	0.324
Weight bearing function recovery G4	0	0.658
	1	0.342

**Table 7:** Ankle joint range of motion. Range of Motion of Ankle Joint: Maximum Physiological Activity and Normal Gait Range for the 3-PUU-R Hybrid Serial–Parallel Rehabilitation Mechanism.

Joint Name	Motion Parameters	Maximum Physiological Activity	Normal Gait Range
Tibiotalar joint	Plantar flexion	0–50°	0–40°
	Dorsiflex	0–30°	0–10°
subtalar joint	Varus	0–25°	0–10°
	Under Pronation	0–15°	0–5°
Coordinated movement of both joints.	Internal Rotation	0–20°	0–5°
	External Rotation	0–10°	0–5°

## Data Availability

The raw data supporting the conclusions of this article will be made available by the authors on request.

## Supplementary Materials

The following supporting information can be downloaded at: https://www.mdpi.com/article/10.3390/biomimetics10120823/s1, Supplementary Material. Randomly allocate disability level data; Table S1: The data on naturally randomized disability levels (without grouping patterns, with cross-distribution of levels).

## Appendix A. Benchmarking Analysis of Rehabilitation Devices

Below is a comparative analysis of key performance metrics across fixed-axis devices, single-degree-of-freedom (DOF) devices, the 3-PUU parallel mechanism (a typical multi-DOF structure), and the proposed 3-PUU-R system integrated with Bayesian Information Gain.

**Table A1:** Performance comparison of fixed-axis, single-DOF, 3-PUU, and proposed 3-PUU-R rehabilitation systems.

Performance Metric	Fixed-Axis Devices	Single-DOF Devices	3-PUU Parallel Mechanism (Typical Multi-DOF Structure)	Proposed System (3-PUU-R + Bayesian IG)
Degrees of Freedom (DOF)	1–2 DOF	1 DOF	3 DOF (2 rotational + 1 translational)	4 DOF (3 rotational + 1 tractional)
ROM Coverage (%)	~40–60% (some ankle movements restricted)	~30–40% (only flexion–extension or single-axis training)	~70–80% (covers most flexion–extension + partial rotation)	>95% (covers full physiological range)
Axis Matching	None (fixed preset axis)	None	Limited (structurally adaptable but no real-time control)	Real-time dynamic matching, automatically aligning with patient’s motion axis
Biomechanical Compatibility	Low, frequent stress concentration and deviation	Low, only supports basic training	Moderate, partially reduces stress but cannot fully prevent axis deviation	High, simulations show reduced stress concentration and closer alignment to natural motion
Control Strategy	Open-loop or fixed program	Passive control	Conventional closed-loop (trajectory tracking)	Closed-loop personalized control driven by Bayesian Information Gain
Intelligence	None	None	Low (depends on simple sensor feedback)	High: IG-based feature selection + Bayesian inference for decision optimization
Real-time Adaptability	None	None	Limited (delayed response)	Strong: training intensity and mode adapt in real time to patient status
Clinical Applicability	Low, only suitable for basic passive rehabilitation	Low, only suitable for early-stage rehabilitation	Moderate, suitable for medium-level training	High, supports full-cycle rehabilitation (acute–recovery–maintenance)
