# Supplementary material for: Development of a Bayesian Network and Information Gain-Based Axis Dynamic Mechanism for Ankle Joint Rehabilitation
Source: Biomimetics (Basel). 2025 Dec 9;10(12):823. doi: 10.3390/biomimetics10120823 (PMC12731023; doi:10.3390/biomimetics10120823)
Supplement: Supplementary file 1 [file biomimetics-10-00823-s001.zip › biomimetics-3953551-supplementary.pdf]

Supplementary Material

Supplementary Material. Randomly allocate disability level data

The following are 40 data points, with the disability levels completely randomly mixed (with no grouping pattern, levels I, II and III appear in a cross-distribution, and special cases are naturally integrated). The features are associated with the levels according to clinical logic, but there is no deliberate correspondence, making the patterns more subtle.

Table S1. The data on naturally randomized disability levels (without grouping patterns, with cross-distribution of levels).

Patient ID	Pain Level (VAS Score)	Swelling Area (Diameter)	Joint Stability (Clinical Examination)	Degree of Activity Limitation	Disability Level	Clinical Scenario Description (Typical/Special)
1	Moderate (4, pain during activity)	Moderate (5cm, slightly elevated skin temperature)	Basically stable (weak positive anterior drawer test)	Moderate (needs handrail for stairs)	Level Ⅱ	Typical case: Partial ligament tear (subacute stage)
2	Mild (1, no pain at rest)	Localized (2cm, normal skin temperature)	Stable (negative lateral stress test)	Mild (normal daily activities)	Level Ⅰ	Typical case: Mild muscle strain
3	Severe (7, persistent pain at rest)	Extensive (9cm, significantly elevated skin temperature)	Unstable (strong positive anterior drawer test)	Severe (requires crutches to walk)	Level Ⅲ	Typical case: Complete anterior cruciate ligament tear (acute stage)
4	Moderate (5, requires intermittent medication)	Localized (3cm, indentation recovery after 5 seconds)	Unstable (II-degree relaxation in lateral stress test)	Severe (unable to stand on one leg)	Level Ⅲ	Special case: Acute medial collateral ligament tear (swelling not yet spread)
5	Mild (2, discomfort after long walks)	Moderate (4cm, normal skin temperature)	Basically stable (no noticeable shaking)	Mild (only limited to fast running)	Level Ⅰ	Typical case: Small joint effusion (non-traumatic)
6	Moderate (4, pain after prolonged sitting)	Moderate (5.5cm, elevated skin temperature)	Basically stable (I-degree relaxation in lateral stress test)	Moderate (difficulty bending forward)	Level Ⅱ	Typical case: Soft tissue edema around the joint
7	Mild (1, almost painless)	Extensive (7cm, slightly elevated skin temperature)	Stable (all tests negative)	Mild (no restriction in activities)	Level Ⅰ	Special case: Chronic synovitis (wide swelling but no structural damage)
8	Severe (8, requires opioid medication)	Extensive (10cm, indentation >30 seconds)	Unstable (unable to bear weight)	Severe (bedridden)	Level Ⅲ	Typical case: Tibial plateau fracture (with severe soft tissue damage)
9	Moderate (5,	Localized (2.5cm,	Unstable (swaying	Severe (needs	Level Ⅲ	Special case:

	worsened pain during activity)	slightly elevated skin temperature)	while walking)	assistance for daily activities)		Posterior cruciate ligament tear (6 hours post-injury, swelling not yet widespread)
10	Mild (2, mild pain when bending the knee too deep)	Moderate (4cm, normal skin temperature)	Stable (negative anterior drawer test)	Mild (able to perform squats)	Level Ⅰ	Typical case: Mild joint capsule injury
11	Moderate (4, significant pain when bearing weight)	Moderate (5cm, normal skin temperature)	Basically stable (negative lateral stress test)	Moderate (can stand on one leg for 20 seconds)	Level Ⅱ	Typical case: Bone contusion (no fracture)
12	Severe (7, pain waking up at night)	Extensive (8cm, high skin temperature)	Unstable (significant swaying)	Severe (needs assistance)	Level Ⅲ	Typical case: Multiple ligament injuries (acute stage)
13	Moderate (5, occasional pain at rest)	Localized (2cm, no indentation)	Stable (walking is unrestricted)	Moderate (only limited by intense activities)	Level Ⅱ	Special case: Moderate meniscus tear (chronic stage, swelling has subsided)
14	Mild (1, discomfort in specific postures)	Localized (1.8cm, normal skin temperature)	Stable (no abnormal shaking)	Mild (normal activity)	Level Ⅰ	Typical case: Mild tendon abrasion
15	Severe (8, intense pain at rest)	Extensive (11cm, swelling spread to the calf)	Unstable (all tests positive)	Severe (completely immobile)	Level Ⅲ	Typical case: Joint dislocation (acute stage)
16	Moderate (4, relief after activity)	Moderate (4.5cm, indentation recovery in 3 seconds)	Basically stable (weak positive anterior drawer test)	Moderate (slow stair climbing)	Level Ⅱ	Typical case: Partial ligament fiber tear (recovery phase)
17	Mild (2, no noticeable pain)	Moderate (3.5cm, slightly elevated skin temperature)	Basically stable (slight swaying when walking fast)	Mild (slight restriction in bending)	Level Ⅰ	Typical case: Mild synovitis
18	Moderate (5, requires ibuprofen for relief)	Localized (3cm, elevated skin temperature)	Unstable (III-degree lateral stress test relaxation)	Severe (needs crutches, unable to climb stairs)	Level Ⅲ	Special case: Complete medial collateral ligament tear (swelling localized but extremely poor stability)
19	Mild (1, mild pain after sitting for long periods)	Extensive (7.5cm, normal skin temperature)	Stable (negative lateral stress test)	Mild (no restriction in daily activities)	Level Ⅰ	Special case: Lymphatic reflux disorder-related

						swelling (no structural damage)
20	Moderate (4, tingling pain at rest)	Moderate (4cm, slightly elevated skin temperature)	Basically stable (negative anterior drawer test)	Moderate (limited deep squats)	Level Ⅱ	Typical case: Mild meniscus injury
21	Severe (7, needs medication for control)	Extensive (8.5cm, significantly elevated skin temperature)	Unstable (unable to stand on one leg)	Severe (needs help getting in and out of bed)	Level Ⅲ	Typical case: Comminuted patella fracture
22	Moderate (5, pain during activity)	Localized (2.5cm, painful upon pressing)	Unstable (strong positive anterior drawer test)	Severe (unable to stand on one leg)	Level Ⅲ	Special case: Anterior cruciate ligament tear (4 hours post-injury, swelling not yet widespread)
23	Mild (2, mild pain after activity)	Moderate (5cm, slightly elevated skin temperature)	Stable (all tests negative)	Mild (no obvious limitations)	Level Ⅰ	Typical case: Mild joint capsule effusion (non-traumatic)
24	Moderate (4, pain when walking with weight)	Moderate (5cm, normal skin temperature)	Basically stable (I-degree relaxation in lateral stress test)	Moderate (can stand on one leg for 15 seconds)	Level Ⅱ	Typical case: Mild ligament tear with contusion
25	Severe (8, significant pain at rest)	Extensive (9cm, significantly elevated skin temperature)	Unstable (requires crutches for walking)	Severe (unable to dress independently)	Level Ⅲ	Typical case: Ankle fracture with ligament tear
26	Mild (1, discomfort only after fast running)	Localized (2cm, no pain upon pressing)	Stable (negative lateral stress test)	Mild (normal daily activities)	Level Ⅰ	Typical case: Mild muscle fatigue
27	Moderate (5, pain when climbing stairs)	Moderate (4.5cm, indentation recovery in 5 seconds)	Basically stable (weak positive anterior drawer test)	Moderate (needs handrail)	Level Ⅱ	Typical case: Mild meniscus tear (subacute phase)
28	Mild (2, mild pain when bending the knee >120°)	Moderate (4cm, normal skin temperature)	Stable (negative anterior drawer test)	Mild (only limited in high-difficulty actions)	Level Ⅰ	Typical case: Mild joint wear (degenerative changes)
29	Severe (7, continuous pain at rest)	Localized (3cm, high skin temperature)	Unstable (significant swaying)	Severe (needs assistance)	Level Ⅲ	Special case: Osteochondral fracture (localized swelling but poor stability)
30	Moderate (4, pain	Moderate (5cm,	Basically stable	Moderate	Level Ⅱ	Typical case:

	during activity)	elevated skin temperature)	(no swaying)	(difficulty climbing stairs)		Moderate soft tissue contusion
31	Mild (1, no pain at rest)	Extensive (7cm, slightly elevated skin temperature)	Stable (negative lateral stress test)	Mild (no limitations in activities)	Level Ⅰ	Special case: Aseptic joint effusion (wide swelling but mild injury)
32	Moderate (5, occasionally needs medication)	Localized (2cm, normal skin temperature)	Unstable (significant swaying when walking)	Severe (unable to stand on one leg)	Level Ⅲ	Special case: Chronic posterior cruciate ligament tear (pain adaptation but function loss)
33	Mild (2, discomfort after long standing)	Moderate (3.5cm, indentation recovery in 2 seconds)	Basically stable (no obvious abnormalities)	Mild (normal daily activities)	Level Ⅰ	Typical case: Mild synovitis (mild symptoms)
34	Moderate (4, occasional pain at rest)	Moderate (5.5cm, slightly elevated skin temperature)	Basically stable (negative anterior drawer test)	Moderate (limited bending)	Level Ⅱ	Typical case: Soft tissue edema around the joint (injurious)
35	Severe (8, pain waking up at night)	Extensive (10cm, indentation >20 seconds)	Unstable (all tests positive)	Severe (bedridden)	Level Ⅲ	Typical case: Multiple ligament injuries in the knee (acute phase)
36	Moderate (5, worsened pain during activity)	Localized (2.5cm, slightly elevated skin temperature)	Unstable (II-degree relaxation in lateral stress test)	Severe (needs crutches, cannot run fast)	Level Ⅲ	Special case: Acute ligament tear (swelling not yet widespread but stability lost)
37	Mild (1, pain only during specific movements)	Localized (1.5cm, normal skin temperature)	Stable (all tests negative)	Mild (can complete squats)	Level Ⅰ	Typical case: Mild joint capsule injury (late recovery)
38	Moderate (4, significant pain when bearing weight)	Moderate (5cm, normal skin temperature)	Basically stable (negative lateral stress test)	Moderate (can stand on one leg for 10 seconds)	Level Ⅱ	Typical case: Bone contusion (with mild edema)
39	Severe (7, requires medication for pain relief)	Extensive (8cm, significantly elevated skin temperature)	Unstable (strong positive anterior drawer test)	Severe (needs assistance dressing)	Level Ⅲ	Typical case: Anterior cruciate ligament tear with meniscus injury
40	Moderate (5 points, occasional stinging pain at rest)	Moderate (4 cm, slightly elevated skin temperature)	Basically stable (anterior drawer test weakly positive)	Moderate (slow when going up and down stairs)	Level II	Typical case: Mild meniscus tear (with synovial inflammation)
41	Mild (2 points, slight pain after	Localized (2.2 cm, normal skin	Stable (negative lateral stress test)	Mild (no limitation in daily activities)	Level Ⅰ	Typical Case: Mild tendonitis (recovery

	walking)	temperature)				phase)
42	Severe (8 points, continuous resting pain)	Extensive (9.5 cm, high skin temperature)	Unstable (unable to stand with weight bearing)	Severe (bedridden, needs assistance for feeding)	Level Ⅲ	Typical Case: Distal femoral fracture (with joint dislocation)
43	Moderate (4 points, pain when getting up after prolonged sitting)	Moderate (4.8 cm, pitting edema resolving in 4 seconds)	Basically Stable (weakly positive anterior drawer test)	Moderate (slight difficulty in bending over)	Level Ⅱ	Typical Case: Partial ligament tear (late subacute phase)
44	Mild (1 point, no obvious pain)	Extensive (6.8 cm, slightly increased skin temperature)	Stable (no abnormal instability)	Mild (only limited in strenuous exercise)	Level Ⅰ	Special Case: Chronic joint effusion (no structural damage)
45	Severe (7 points, awakened by pain multiple times at night)	Extensive (10.5 cm, swelling to ankle)	Unstable (all tests strongly positive)	Severe (complete inability to get out of bed)	Level Ⅲ	Typical Case: Comminuted ankle fracture (acute phase)
46	Moderate (5 points, intermittent painkiller use required)	Localized (3.2 cm, increased skin temperature)	Unstable (Level Ⅲ laxity in lateral stress test)	Severe (unable to stand on one leg for more than 3 seconds)	Level Ⅲ	Special Case: Complete medial collateral ligament tear (3 hours after injury, localized swelling)
47	Mild (2 points, slight pain when knee is flexed deeply)	Moderate (3.8 cm, normal skin temperature)	Basically Stable (no instability when walking fast)	Mild (normal daily activities)	Level Ⅰ	Typical Case: Small amount of joint effusion (physiological)
48	Moderate (4 points, pain relieved after activity)	Moderate (5.2 cm, slightly increased skin temperature)	Basically Stable (Level Ⅰ laxity in lateral stress test)	Moderate (slow when going up/down stairs)	Level Ⅱ	Typical Case: Mild periarticular soft tissue edema
49	Severe (8 points, opioid medications required for pain relief)	Extensive (10.2 cm, pitting edema lasting >25 seconds)	Unstable (obvious unsteady feeling when walking)	Severe (needs bilateral crutches, unable to stand on one leg)	Level Ⅲ	Typical Case: Comminuted tibial plateau fracture (with soft tissue necrosis)
50	Mild (1 point, discomfort only in specific postures)	Localized (1.9 cm, normal skin temperature)	Stable (all tests negative)	Mild (able to complete daily movements)	Level Ⅰ	Typical Case: Minor muscle strain (early recovery phase)
51	Moderate (5 points, pain when walking with weight bearing)	Moderate (4.6 cm, normal skin temperature)	Basically Stable (negative lateral stress test)	Moderate (able to stand on one leg for 18 seconds)	Level Ⅱ	Typical Case: Bone contusion (no obvious edema)
52	Severe (7 points,	Extensive (8.2 cm,	Unstable (strongly	Severe (needs	Level Ⅲ	Typical Case:

	obvious resting pain)	significantly increased skin temperature)	positive anterior drawer test)	assistance for getting in/out of bed)		Patellar dislocation combined with ligament tear (acute phase)
53	Mild (2 points, discomfort after prolonged standing)	Localized (2.3 cm, no pitting edema)	Stable (walks freely)	Mild (no limitation in daily activities)	Level Ⅰ	Typical Case: Mild synovitis (mild symptoms)
54	Moderate (4 points, pain worsening during activity)	Moderate (5.1 cm, increased skin temperature)	Basically Stable (negative anterior drawer test)	Moderate (significant limitation in deep squat)	Level Ⅱ	Typical Case: Moderate meniscus injury (subacute phase)
55	Severe (8 points, severe pain at night)	Extensive (11.2 cm, swelling diffused to thigh)	Unstable (unable to turn over independently)	Severe (completely dependent on others for care)	Level Ⅲ	Typical Case: Hip dislocation (with acetabular fracture)
56	Mild (1 point, slight pain after prolonged sitting)	Extensive (7.2 cm, normal skin temperature)	Stable (negative lateral stress test)	Mild (no activity limitation)	Level Ⅰ	Special Case: Lymphedema (chronic phase, stable symptoms)
57	Moderate (5 points, needs ibuprofen for pain relief)	Localized (3.1 cm, slightly increased skin temperature)	Unstable (Level Ⅱ laxity in lateral stress test)	Severe (needs crutches, unable to go up/down stairs)	Level Ⅲ	Special Case: Posterior cruciate ligament tear (5 hours after injury, swelling not diffused)
58	Mild (2 points, no resting pain)	Moderate (3.6 cm, pitting edema resolving in 2 seconds)	Basically Stable (no obvious abnormality)	Mild (only slight limitation in sprinting)	Level Ⅰ	Typical Case: Minor joint capsule injury (early phase)
59	Moderate (4 points, occasional pain at rest)	Moderate (5.3 cm, slightly increased skin temperature)	Basically Stable (weakly positive anterior drawer test)	Moderate (limited in bending over)	Level Ⅱ	Typical Case: Periarticular soft tissue edema (early injury phase)
60	Severe (7 points, medication required for pain control)	Extensive (8.8 cm, high skin temperature)	Unstable (obvious instability)	Severe (needs assistance for daily activities)	Level Ⅲ	Typical Case: Multiple ligament injuries (subacute phase)
61	Mild (1 point, slight pain only after activity)	Localized (1.7 cm, normal skin temperature)	Stable (all tests negative)	Mild (able to complete deep squat)	Level Ⅰ	Typical Case: Minor tendon wear (mild)
62	Moderate (5 points, obvious pain when bearing weight)	Moderate (4.9 cm, normal skin temperature)	Basically Stable (negative lateral stress test)	Moderate (able to stand on one leg for 12 seconds)	Level Ⅱ	Typical Case: Bone contusion (with mild synovitis)

63	Severe (8 points, severe resting pain)	Extensive (9.8 cm, significantly increased skin temperature)	Unstable (unable to stand on one leg)	Severe (needs assistance for washing)	Level Ⅲ	Typical Case: Knee dislocation (with cartilage injury)
64	Mild (2 points, slight pain when knee is flexed >90°)	Extensive (7.1 cm, slightly increased skin temperature)	Stable (no unsteady feeling)	Mild (normal daily activities)	Level Ⅰ	Special Case: Aseptic joint effusion (chronic phase)
65	Moderate (4 points, pain during activity)	Localized (2.8 cm, slightly increased skin temperature)	Basically Stable (negative anterior drawer test)	Moderate (needs handrail for going up/down stairs)	Level Ⅱ	Typical Case: Minor ligament tear (recovery phase)
66	Severe (7 points, awakened by pain at night)	Extensive (8.5 cm, pitting edema lasting >15 seconds)	Unstable (Level Ⅲ laxity in lateral stress test)	Severe (needs crutches, unable to walk more than 10 meters)	Level Ⅲ	Typical Case: Medial collateral ligament tear combined with meniscus tear
67	Mild (1 point, no obvious pain)	Localized (2.1 cm, normal skin temperature)	Stable (negative lateral stress test)	Mild (no activity limitation)	Level Ⅰ	Typical Case: Minor muscle strain (late recovery phase)
68	Moderate (5 points, occasional stabbing pain at rest)	Moderate (4.7 cm, pitting edema resolving in 3 seconds)	Basically Stable (weakly positive anterior drawer test)	Moderate (limited in bending over)	Level Ⅱ	Typical Case: Mild meniscus injury (no obvious edema)
69	Severe (8 points, medication required for pain relief)	Extensive (10.8 cm, swelling to mid-calf)	Unstable (all tests positive)	Severe (bedridden, unable to turn over independently)	Level Ⅲ	Typical Case: Proximal tibial fracture (with severe soft tissue contusion)
70	Mild (2 points, discomfort after walking)	Moderate (3.9 cm, normal skin temperature)	Stable (no abnormal instability)	Mild (only limited in strenuous exercise)	Level Ⅰ	Typical Case: Mild synovitis (recovery phase)
71	Moderate (4 points, pain when getting up after prolonged sitting)	Localized (3.3 cm, increased skin temperature)	Basically Stable (Level Ⅰ laxity in lateral stress test)	Moderate (able to stand on one leg for 15 seconds)	Level Ⅱ	Typical Case: Periarticular soft tissue edema (chronic phase)
72	Severe (7 points, continuous resting pain)	Extensive (9.2 cm, high skin temperature)	Unstable (strongly positive anterior drawer test)	Severe (needs assistance for dressing)	Level Ⅲ	Typical Case: Complete anterior cruciate ligament tear (acute exacerbation of chronic phase)
73	Mild (1 point, pain only in specific movements)	Extensive (6.9 cm, slightly increased skin temperature)	Stable (all tests negative)	Mild (able to complete deep squat)	Level Ⅰ	Special Case: Chronic synovitis (mild symptoms)
74	Moderate (5	Moderate (5.4 cm,	Basically Stable	Moderate (slow	Level Ⅱ	Typical Case: Partial

	points, pain relieved after activity)	slightly increased skin temperature)	(negative anterior drawer test)	when going up/down stairs)		ligament tear (recovery phase)
75	Severe (8 points, obvious resting pain)	Extensive (11.5 cm, swelling diffused to lower thigh)	Unstable (unable to bear weight)	Severe (bedridden, needs nasogastric feeding)	Level Ⅲ	Typical Case: Comminuted hip fracture (with nerve injury)
76	Mild (2 points, no obvious pain)	Localized (2.4 cm, normal skin temperature)	Stable (negative lateral stress test)	Mild (normal daily activities)	Level Ⅰ	Typical Case: Minor tendon injury (early phase)
77	Moderate (4 points, pain when walking with weight bearing)	Moderate (4.5 cm, pitting edema resolving in 5 seconds)	Basically Stable (negative lateral stress test)	Moderate (difficulty in bending over)	Level Ⅱ	Typical Case: Bone contusion (acute phase)
78	Severe (7 points, medication required for pain control)	Extensive (8.7 cm, significantly increased skin temperature)	Unstable (Level Ⅱ laxity in lateral stress test)	Severe (needs crutches, unable to get in/out of bed)	Level Ⅲ	Typical Case: Ankle ligament tear combined with fracture
79	Mild (1 point, slight pain after walking)	Extensive (7.3 cm, normal skin temperature)	Stable (no unsteady feeling)	Mild (only limited in sprinting)	Level Ⅰ	Special Case: Lymphedema (recovery phase)
80	Moderate (5 points, pain worsening during activity)	Moderate (5.3 cm, normal skin temperature)	Basically Stable (weakly positive anterior drawer test)	Moderate (able to stand on one leg for 8 seconds)	Level Ⅱ	Typical Case: Moderate meniscus tear (recovery phase)
81	Severe (8 points, awakened by pain multiple times at night)	Extensive (10.3 cm, pitting edema lasting >20 seconds)	Unstable (all tests strongly positive)	Severe (needs assistance for feeding)	Level Ⅲ	Typical Case: Multiple knee ligament injuries (chronic phase)
82	Mild (2 points, slight pain when knee is flexed deeply)	Localized (1.8 cm, normal skin temperature)	Stable (all tests negative)	Mild (able to complete daily movements)	Level Ⅰ	Typical Case: Minor joint capsule injury (early phase)
83	Moderate (4 points, occasional pain at rest)	Moderate (4.2 cm, slightly increased skin temperature)	Basically Stable (Level Ⅰ laxity in lateral stress test)	Moderate (slow when going up/down stairs)	Level Ⅱ	Typical Case: Minor ligament tear (subacute phase)
84	Severe (7 points, severe resting pain)	Extensive (9.6 cm, high skin temperature)	Unstable (unable to stand on one leg)	Severe (needs bilateral crutches, unable to walk)	Level Ⅲ	Typical Case: Comminuted patellar fracture (with ligament injury)
85	Mild (1 point, discomfort only in specific postures)	Extensive (7.5 cm, slightly increased skin temperature)	Stable (no abnormal instability)	Mild (no limitation in daily activities)	Level Ⅰ	Special Case: Joint effusion (non-traumatic)

86	Moderate (5 points, intermittent painkiller use required)	Localized (2.9 cm, increased skin temperature)	Basically Stable (negative anterior drawer test)	Moderate (limited in bending over)	Level Ⅱ	Typical Case: Mild periarticular soft tissue contusion
87	Severe (8 points, opioid medications required)	Extensive (8.3 cm, significantly increased skin temperature)	Unstable (strongly positive anterior drawer test)	Severe (needs assistance for washing)	Level Ⅲ	Typical Case: Anterior cruciate ligament tear combined with cartilage injury
88	Mild (2 points, no resting pain)	Moderate (3.7 cm, normal skin temperature)	Stable (negative lateral stress test)	Mild (only limited in strenuous exercise)	Level Ⅰ	Typical Case: Minor muscle strain (early phase)
89	Moderate (4 points, obvious pain when bearing weight)	Moderate (5.0 cm, pitting edema resolving in 3 seconds)	Basically Stable (negative lateral stress test)	Moderate (able to stand on one leg for 13 seconds)	Level Ⅱ	Typical Case: Bone contusion (with synovitis)
90	Severe (7 points, medication required for pain relief)	Extensive (9.9 cm, high skin temperature)	Unstable (Level Ⅲ laxity in lateral stress test)	Severe (needs crutches, unable to sprint)	Level Ⅲ	Typical Case: Complete medial collateral ligament tear (chronic phase)
91	Mild (1 point, slight pain after activity)	Localized (2.6 cm, normal skin temperature)	Stable (all tests negative)	Mild (able to complete deep squat)	Level Ⅰ	Typical Case: Mild tendonitis (chronic phase)
92	Moderate (5 points, pain when getting up after prolonged sitting)	Moderate (4.4 cm, slightly increased skin temperature)	Basically Stable (weakly positive anterior drawer test)	Moderate (needs handrail for going up/down stairs)	Level Ⅱ	Typical Case: Partial ligament tear (chronic phase)
93	Severe (8 points, continuous resting pain)	Extensive (10.6 cm, swelling to upper calf)	Unstable (unable to stand with weight bearing)	Severe (bedridden, needs assistance for turning over)	Level Ⅲ	Typical Case: Tibial plateau fracture (with intra-articular hemorrhage)
94	Mild (2 points, no obvious pain)	Extensive (7.6 cm, normal skin temperature)	Stable (no unsteady feeling)	Mild (normal daily activities)	Level Ⅰ	Special Case: Chronic synovitis (stable phase)
95	Moderate (4 points, pain relieved after activity)	Localized (3.4 cm, slightly increased skin temperature)	Basically Stable (Level Ⅰ laxity in lateral stress test)	Moderate (slight difficulty in bending over)	Level Ⅱ	Typical Case: Periarticular soft tissue edema (recovery phase)
96	Severe (7 points, awakened by pain at night)	Extensive (8.9 cm, significantly increased skin temperature)	Unstable (obvious instability)	Severe (needs assistance for daily activities)	Level Ⅲ	Typical Case: Multiple ligament injuries (late acute phase)
97	Mild (1 point, pain	Moderate (3.5 cm,	Stable (negative	Mild (only limited	Level Ⅰ	Typical Case: Minor

	only in specific movements)	normal skin temperature)	lateral stress test)	in sprinting)		joint capsule injury (chronic phase)
98	Moderate (5 points, occasional stabbing pain at rest)	Moderate (4.3 cm, pitting edema resolving in 4 seconds)	Basically Stable (negative anterior drawer test)	Moderate (able to stand on one leg for 11 seconds)	Level Ⅱ	Typical Case: Mild meniscus injury (chronic phase)
99	Severe (8 points, obvious resting pain)	Extensive (11.1 cm, swelling diffused to mid-thigh)	Unstable (all tests positive)	Severe (needs assistance for dressing)	Level Ⅲ	Typical Case: Hip dislocation (with ligament tear)
100	Moderate (4 points, pain when walking with weight bearing)	Moderate (4.1 cm, slightly increased skin temperature)	Basically Stable (weakly positive anterior drawer test)	Moderate (slow when going up/down stairs)	Level Ⅱ	Typical Case: Bone contusion (recovery phase)
